# Porphyrins and Phthalocyanines on Solid-State Mesoporous Matrices as Catalysts in Oxidation Reactions

**DOI:** 10.3390/ma15072532

**Published:** 2022-03-30

**Authors:** Joanna Szymczak, Michal Kryjewski

**Affiliations:** Chair and Department of Inorganic and Analytical Chemistry, Poznan University of Medical Sciences, Rokietnicka 3, 60-806 Poznan, Poland; j.nowicka@gmail.com

**Keywords:** catalysis, mesoporous silica, oxidation, phthalocyanine, porphyrin

## Abstract

The review presents recent examples of heterogenic catalysts based on porphyrins and phthalocyanines loaded on mesoporous materials, such as MCM-41, SBA-15, MCM-48, SBA-16 or Al-MCM-41. Heterogenic approach to catalysis eases recovery, reuse and prevent macrocycle aggregation. In this application, mesoporous silica is a promising candidate for anchoring macrocycle and obtaining a new catalyst. Introduction of porphyrin or phthalocyanine into the mesoporous material may be performed through adsorption of the macrocycle, or by its in situ formation—by reaction of substrates introduced to the pores of the catalytic material. Catalytic reactions studied are oxidation processes, focused on alkane, alkene or arene as substrates. The products obtained are usually epoxides, alcohols, ketones, aldehydes or acids. The greatest interest lies in oxidation of cyclohexane and cyclohexene, as a source of adypic acid and derivatives. Some of the reactions may be viewed as biomimetic processes, resembling processes that occur in vivo and are catalyzed by cytochrome P450 enzyme family.

## 1. Introduction

Macrocyclic tetrapyrroles are aromatic heterocyclic compounds from among the best known are porphyrins and chlorins. They play an important role in living processes, as they are prosthetic groups in many biologically active enzymes and proteins. The most important groups of naturally occurring chlorin derivatives are chlorophylls, as they show light-harvesting properties [1]. Similarly, heme is crucial for living organisms, as it is responsible for storage and transport of oxygen, and it also forms an active center of cytochrome P-450 [2,3]. Apart from porphyrins there are other numerous synthetic tetrapyrrole macrocycles, such as phthalocyanines (Pcs), chlorins, corroles and porphyrazines [4,5,6,7]. Applications of porphyrins, phthalocyanines and related macrocycles are intensively studied in many fields. Apart from their traditional use as dyes, macrocycles are used as photosensitizers for photodynamic therapy both in free form [8,9] or as a part of nanoparticles [10,11]. Moreover, tetrapyrrolic macrocycles are applied in optical filters or solar cells [5,12,13], and as catalysts [14,15]. As these molecules resemble naturally occurring porphyrins and derivatives in both structure and function, their catalytic and photocatalytic properties are constantly investigated. Macrocycles may be used to mimic the activity of cytochrome P450 in selective oxidation of organic molecules, leading to valuable chemicals [16,17]. In a more robust approach, they are investigated as a tool for water treatment through the oxidative decomposition of organic pollutants [18,19]. Upon illumination with light, certain macrocycles may generate reactive oxygen species, including singlet oxygen, which further extends the scope of the catalytic activity. Apart from oxidation reactions, the catalytic activity of macrocycles also embraces the formation of cyclic carbonates from epoxides or coupling reaction of diazo compounds, etc.

Because of their structural similarity to heme, tetrapyrrolic macrocycles are promising catalysts in biomimetic reactions, as they can mimic metabolic hydroxylation reactions of xenobiotics. Catalytic reactions in which they are involved can bring benefits in metabolism research, in particular, they are expected to permit development of easy ways to obtain metabolites [20]. Because of importance of biological processes in which porphyrins and derivatives are involved, they are the subject of continuous interest [6]

The search for new catalyst to be used in selective reactions, e.g., oxidation of aromatic hydrocarbons, has led to increased interest in macrocyclic compounds. Due to their high thermal and chemical stability, low cost of synthesis they can be used as catalysts. Catalytic activity of metalated tetrapyrrole macrocycle mainly depends on the electronic structure of metal ion in the center. Hence it is important to choose a right metal for the specific reaction. Immobilization of tetrapyrrole macrocycles on the insoluble supports can enhance the catalyst stability, selectivity and their activity as well as make the catalyst easier to handle and separate from the reaction medium [21].

After the discovery of mesoporous silicas by Mobil group [22] the synthesis, characterization and applications of the solid-state matrices have been of widespread interest in material science. Ordered mesoporous materials are extensively used as excellent host materials because of their unique properties such as controllable pore size, high surface area and stable structure. They are able to encapsulate the molecules into the pores and at the same time allow the solvent, ions and other small molecules into their interior through the channels. One of the best-known mesoporous silicas is MCM-41 (Mobil Composition of Matter No. 41) discovered by the aforementioned Mobil group. It has regular, hexagonal arrays of uniform channels whose dimension can be tailored (from 16 Å to 100 Å or more) through the choice of surfactant, additional chemicals and reaction conditions. Another material from M41S group discovered in 1995 by Bagshaw et al. was the so-called MSU-1 (Michigan State University, Michigan, MI, USA) exhibiting a 3D-wormhole structure [23,24]. Next came the SBA (Santa Barbara Amorphous) family of mesoporous silicas which are amorphous, structurally well-ordered materials with tunable large pore, of the size up to 300 Å. It has cylindrical hexagonal pores, thermally stable structure and large surface area which makes it a promising catalyst [25,26].

There is constant interest in catalytic properties of porphyrins and phthalocyanines [27,28]. Combining their unique properties with mesoporous materials is a subject of ongoing research and review papers [29]. In contrast, to bulk heterogenic catalysts, which contain many poorly characterized active sites [30], there is an ongoing research on catalysts with well-defined active sites. This may be addressed by single atom catalysts (SAC), which have single metal atoms attached to a support [31]. Porphyrins may be utilized in SAC as a source of the evenly-spread metal atoms; macrocycle may be used a substrate in pyrolysis reaction resulting in well-dispersed molecular CoN_x_ sites on the carbon support [32]. In different approach porphyrins are one of the building blocks of metal-organic framework (MOF). In the MOF a porphyrin molecule is intact and becomes a part of 3D structure, capable of catalytic action [33]. While SAC often are focused on the processing of the small molecules such as CO_2_ reduction [30], ammonia synthesis [34] and O_2_ reduction [35], while organic reactions are studied to a lesser extent [31].

This review paper is focused on mesoporous materials, mainly SBA-15 and MCM-41 and covers published research since seminal papers on MCM-41-bound porphyrins by Liu et al. [36] and phthalocyanines by Ernst and Selle [37]. Earlier article concerning mainly zeolites and clays may be useful for reader [38]. We hope the presented article will inspire further research.

## 2. Methods of Immobilization Porphyrins and Phthalocyanines into Mesoporous Materials

**Figure 1:**
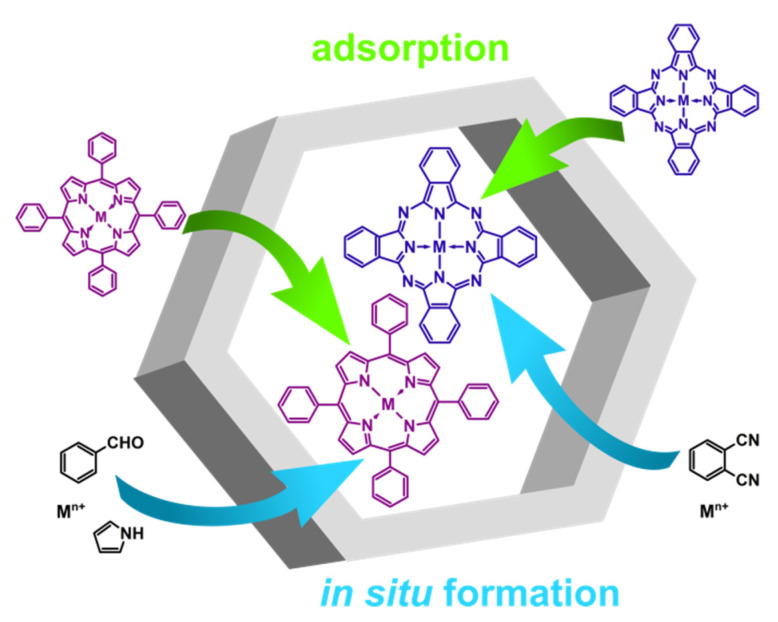
Two methods of immobilization of porphyrins and phthalocyanines into mesoporous materials; adsorption and in situ formation.

Porphyrins and phthalocyanines may be incorporated into mesopores by in situ process or adsorption (Figure 1). Frequently an anchoring group is employed. Adsorption on the surface of mesoporous material takes place through stirring mesoporous material and the macrocycle in solvent able to dissolve the macrocycle. Although there are several methods of immobilization this one is the simplest. To purify the complex obtained filtration and washing with an appropriate solvent is required, followed by drying the solid. The amount of the complex bonded to the mesoporous material can be evaluated on the basis of comparison of UV-Vis spectra of the solution recorded before and after the immobilization. Although this method is simple and does not require the modification of the support and catalyst it has its limitations. Layering and stacking of phthalocyanine can occur while using concentrated solution what prevents the access to the active site of the catalyst. It has to be taken into account while choosing this method.

**Figure 2:**
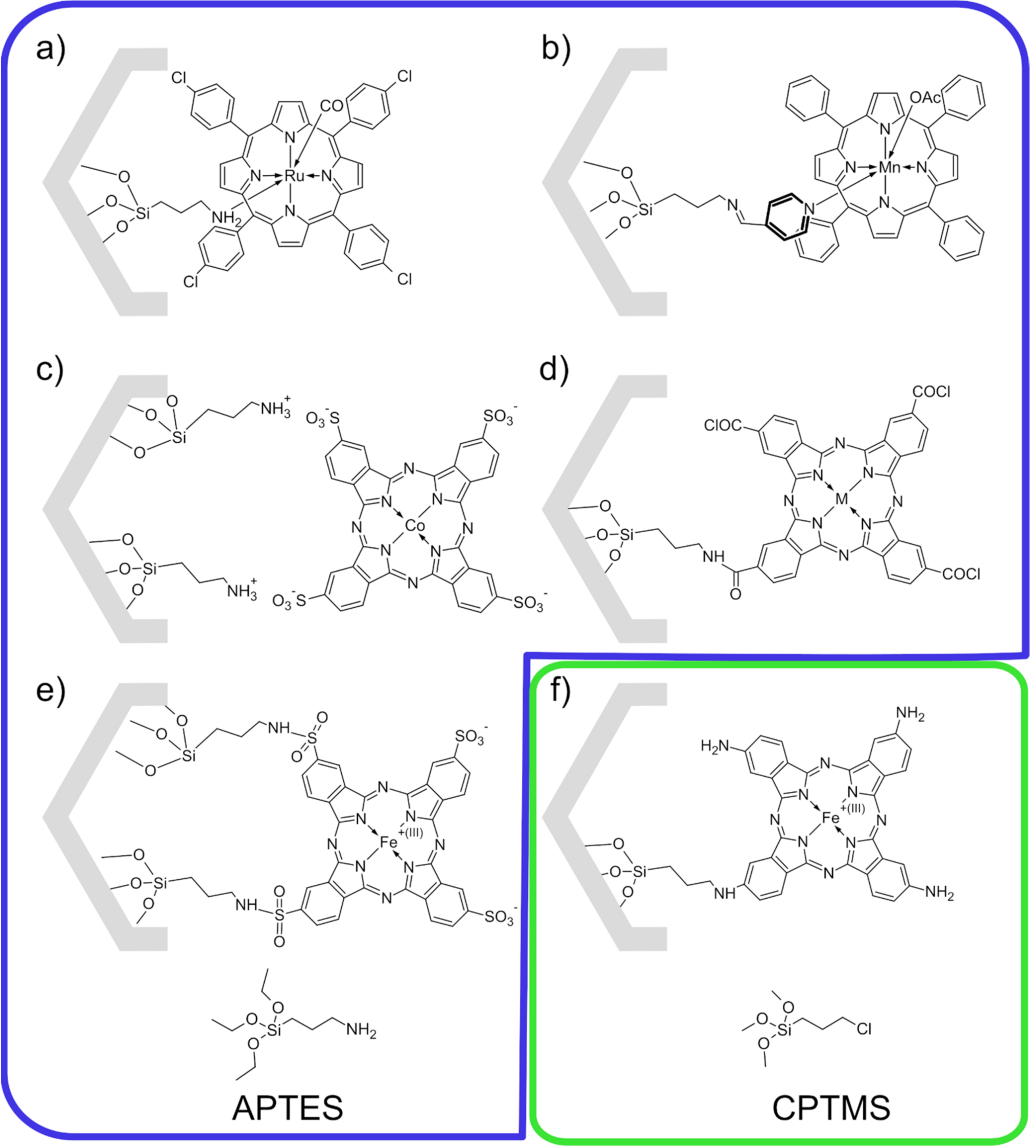
Porphyrins and phthalocyanines incorporated into mesoporous support modified with APTES (3-aminopropyltriethoxysilane)—(**a**) [36], (**b**) [39], (**c**) [40], (**d**) [41] (M = 2H, Zn, Cu, Ni, Co, Fe, Mn) and (**e**) [42] and with CPTES (3-chloropropyltriethoxysilane)—(**f**) [42].

To overcome this drawback, surface of the mesopores may be modified with silylating groups such as 3-aminopropyltriethoxysilane (APTES) or 3-chloropropyltrimethoxysilane (CPTMS), which are commercially available. Various strategies of the using anchoring group are utilized (Figure 2). Liu and co-workers [36] have tried to synthesize a stable complex of ruthenium(II) 5,10,15,20-tetrakis(4-chlorophenyl)porphyrin grafted onto MCM-41. Their first attempt of direct immobilization was unsuccessful because of weak interactions between the porphyrin and MCM-41. To resolve this problem the authors functionalized the surface of MCM-41 with APTES, which permits formation of coordinating bonds between porphyrin molecules and amino groups. After this modification MCM-41 exhibited a powerful encapsulation ability (Figure 2a).

In another study, MCM-48 material was first modified with APTES in order to introduce -NH2 groups, which were further reacted with pyridine-4-carbaldehyde leading to enamine linker (Figure 2b, [39]). The immobilization process was achieved by the complexation reaction between pyridine moiety on the surface of MCM-48, with manganese ion placed in the core of porphyrin. APTES was also used to modify MCM-41 material and subsequently used to bind cobalt tetrasulfophthalocyanine (Figure 2c, [40]). The phthalocyanine loading was conducted in solution of pH = 3, which converted APTES to its cationic form, enabling interaction with the sulfonated phthalocyanine. Different approach is the covalent binding of macrocycles to APTES-modified mesoporous silica. MCM-41 decorated with APTES groups were reacted with a series of tetracarboxylphthalocyanines, converted to acyl chlorides (Figure 2d) resulting in formation of an amide group [41]. In similar manner, iron(III) tetrasulfophthalocyanine was converted to sulfonyl chloride and reacted with APTES-modified MCM-41 (Figure 2e) [42]. The same study describes analogic reaction of MCM-41 modified with CPTMS, which was further reacted with iron(III) tetraminophthalocyanine (Figure 2f).

**Figure 3:**
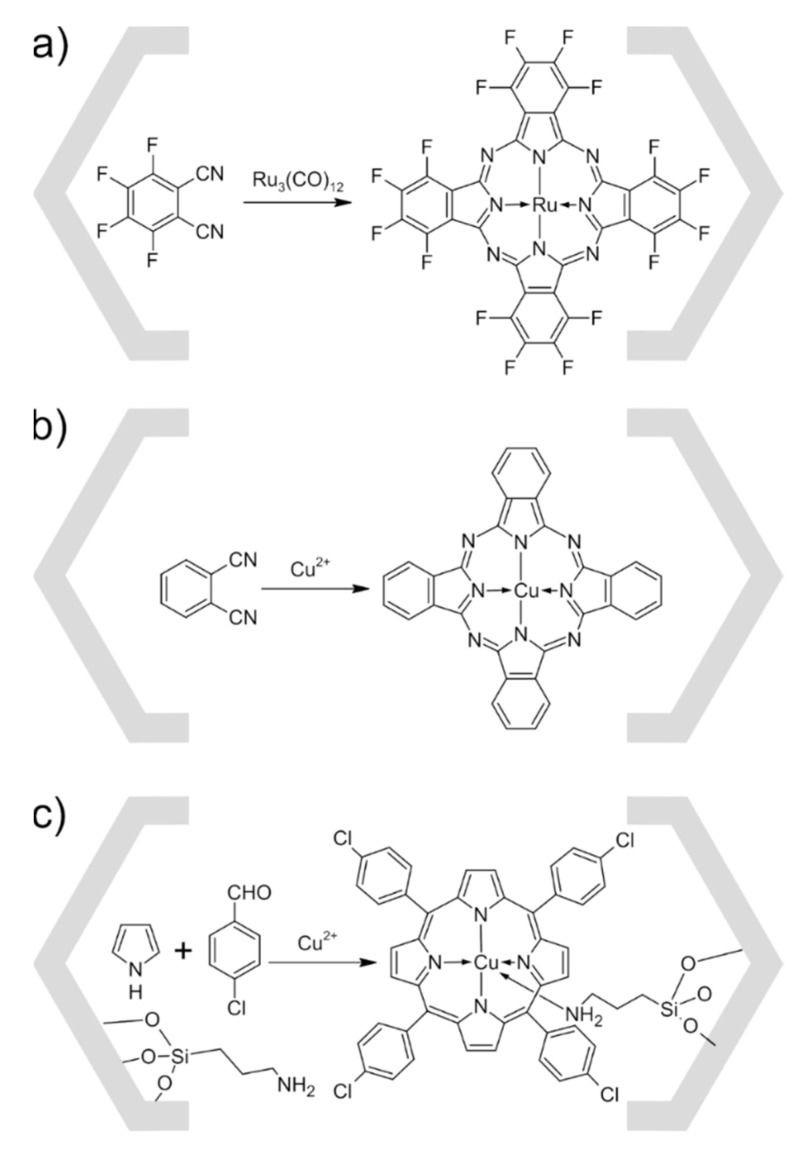
“Ship-in-a-bottle” method of synthesis macrocycles inside mesopores: (**a**) perfluorinated ruthenium(II) phthalocyanine [37], (**b**) copper(II) phthalocyanine [43] and (**c**) copper(II) 5,10,15,20-tetrakis(4-chlorophenyl)porphyrin anchored to APTES molecule [44].

Ernst and Selle [37] explored three different methods for the immobilization of perfluorinated ruthenium phthalocyanine complexes in the pores of MCM-41: (i) the “ship-in-a-bottle” method, (ii) synthesis method and (iii) grafting the complex onto the functionalized surface of mesoporous material. The first method, “ship-in-a-bottle” (Figure 3a) required introduction of ruthenium carbonyl complex and tetrafluorophthalonitrile into the mesoporous material, subsequent heating of the mixture, followed by extensive extraction to remove unreacted materials. This procedure resulted in the formation of phthalocyanine inside the mesopores. However, only the material with smallest pore size (ca. 1.5 nm) retained phthalocyanine after extraction, and more than 90% of the phthalocyanine was in the metal-free form. The second method was an attempt to introduce RuPc into mesoporous material during MCM-41 formation. Ruthenium perfluorophthalocyanine was added to the surfactant solution used for MCM-41 synthesis. Instead of typical calcination process, which is used in mesoporous materials production for removal of the organic template chemicals, extraction with HCl solution in ethanol was applied. Only the material with narrowest pores (~1.9 nm) showed complexed ruthenium phthalocyanine after the work-up. The third method was based on functionalization of mesoporous material. MCM-41 was modified with APTES, followed by incorporation of RuPc and extraction. The authors tested the catalyst prepared according to (i) and (iii) methods for their ability to catalyze the oxidation of n-hexane and cyclohexane, respectively. The material prepared via in situ synthesis was not included in the catalytic studies because of its low complex content. The catalyst prepared after grafting the complex onto the functionalized surface shows low activity which can be attributed to high loading of the mesoporous material with complexes. It can cause partial blockage of the pores. The other possibility is that the Ru-N bonds may modify the nature of the active site. From among these three methods of immobilization only the “ship-in-a-bottle” synthesis provides material with significant catalytic activity.

Chemical vapor deposition with use of 1,2-dicyanobenzene (DCB) was tested [43] in synthesis of copper(II) phthalocyanine (CuPc), which seems to be an effective method because DCB molecules are smaller than the CuPc molecules and can be easily introduced deep into the mesopores of MCM-41 (Figure 3b). Cu^2+^ ions were fixed in the pores by ion exchange, followed by sublimation of DCB, which diffused into the mesopores. Reaction of copper ions and DCB led to CuPc formation. Attempts to sublime CuPc into MCM-41 mesopores resulted in pale-blue material formation. Similar method was used by Pirouzumand et al. [21] who utilized microwave irradiation to immobilize cobalt, manganese and iron phthalocyanines into the mesopores of MCM-41. The mesoporous material MCM-41 was chosen because it contains a silanol-rich surface whose silanol protons can be replaced by metal ions. At first, metal ions (Co^2+^, Mn^2+^, Fe^2+^) were introduced into the pores with the use of ion exchange. Then the obtained metal ion-modified MCM-41 material was mixed with DCB. Microwave irradiation of the mixture resulted in formation of Pc and was followed by washing the material to remove excess of unreacted DCB and Pc. The prepared materials were characterized by UV-Vis spectroscopy, X-ray powder diffraction, differential scanning calorimetry and BET nitrogen adsorption desorption techniques. The results confirmed the formation of metallophthalocyanines into the pore channels of MCM-41. The advantage of this method is a short period of time (in the range of minutes) required for formation of the phthalocyanines.

An efficient and easy method to incorporate the metallophthalocyanines into the mesoporous matrix via an in situ reaction between SBA-15 pre-loaded with metal ions and DCB, precursor of Pc [45]. The metal ions were introduced by two different routes: (i) vacuum-casting method under ultrasonication or (ii) modification of SBA-15 with ethylenediamine moiety, which complexed metal ions. Ni^2+^ and Co^2+^ were used in method (i), while Cu^2+^, Zn^2+^, Ni^2+^ and Co^2+^ in method (ii). MCM-41 materials obtained were mixed with DCB, and heated at 220 °C–250 °C under vacuum, which resulted in sublimation of DCB into the mesopores, and reaction with the pre-dispersed metal ions to form metallophthalocyanine. The phthalocyanines are not directly loaded into the pores. In this case the pore channels are used as “micro-reactors” in which the precursors of metallophthalocyanines (metal ions and DCB molecules) are introduced stepwise to produce incorporated MPcs by in situ reactions.

Liu and co-workers [36] have tried to synthesize a stable complex of ruthenium(II) meso-tetrakis(4-chlorophenyl)porphyrin grafted onto MCM-41. Their first attempt of direct immobilization was unsuccessful because of weak interactions between the porphyrin and MCM-41. To resolve this problem the authors functionalized the surface of MCM-41 with 3-aminopropyltriethoxysilane (APTES), which permits formation of coordinating bonds between porphyrin molecules and amino groups. After this modification MCM-41 exhibited a powerful encapsulation ability.

In another approach [46] NaY zeolite and MCM-41 were used for iron(III) porphyrins incorporation using different methods, and screened for the best catalytic system. In the first attempt, the authors loaded HY zeolite and Al-MCM-41 with ferric nitrate. These metal exchanged materials were loaded with meso-tetraphenyl porphyrin and 5,10,15,20-tetrakis-(4-pyridyl)porphyrin, which subsequently reacted to their respective ferric forms inside the mesopores. Much broader studies were conducted for the “ship-in-a-bottle” method; metal exchanged HY zeolite or Al-MCM-41 were subjected to pyrrole and benzaldehyde derivative, which resulted in porphyrin formation. Six porphyrins were introduced into the mesopores using this manner, 5,10,15,20-tetraphenyl-porphyrin was obtained with highest yield. Additionally, porphyrins were introduced during the zeolite synthesis and anchored by the 3-aminopropyl trimethoxysilane (APTMS) linker, however no details of this procedures were given.

Copper(II) 5,10,15,20-tetrakis(4-chlorophenyl)porphyrin was introduced into mesopores of SBA-16 modified with APTES (Figure 3c) [44]. It was achieved by reacting 4-chlorobenzaldehyde, pyrrole and copper(II) acetate, in the presence of SBA-16. Mesoporous material was washed by soxhlet-extraction with dichloromethane, acetonitrile and chloroform, and subjected to characterization.

## 3. Porphyrins and Phthalocyanines Immobilized on Mesoporous Silicas as Catalysts

The synthesis of new catalysts which can mimic the action of P-450 enzymes has attracted a considerable attention in catalytic chemistry. Synthetic metalloporphyrins, especially iron porphyrins have been successfully used in many oxidation reactions as biomimetic catalysts. Tetrapyrrole macrocycles containing metal ion in their cores can effectively catalyze C-H bond, C-O (hydroxylation), C-N (amination) and C-C bond formation (carbine insertion) as they display peroxidase and oxidase activity. Immobilization of a catalyst onto a solid support is a good way to overcome the typical problems of homogenous catalysis which are formation of inactive aggregates or blockage of the reagents access to the catalytically active sites. Immobilization prevents catalyst from self-destruction, increases stability, facilitates reactants access to the catalyst active sites and enables its reuse [47].

One of the first manganese-porphyrin complex with silica was obtained by Battioni et al. [48]. These authors described the efficacy and effects of a silica support for alkane hydroxylation in the presence of oxygen atom donors such as PhIO. The results were promising and in following years other researchers started to investigate the catalytic activity of porphyrins and phthalocyanines metallic complexes with various mesoporous materials.

### 3.1. Oxidation of Alkanes

Alkanes as little reactive compounds require a large portion of energy to carry out the oxidation reaction. These reactions are important mainly in the chemical industry. Due to the importance of the products of oxidation it is challenging to develop effective catalysts that allow the reaction to be carried out in an environmentally friendly manner. Naturally occurring enzymes which belongs to monooxygenase family such as methane monooxygenase found in living organisms inspire many researchers to design new catalyst [49,50].

#### 3.1.1. Porphyrin-Based Catalysts

Porphyrins were the first macrocyclic compounds immobilized on mesoporous materials. At the beginning zeolites were employed for the encapsulation. However, there have small pore size make difficulties in immobilization of tetrapyrrole macrocycles. Thus, more advantageous materials—with larger size pores such as MCM-41—were taken into consideration.

**Scheme 1:**
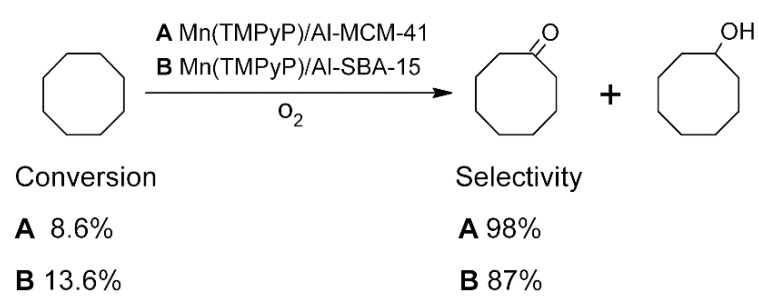
Oxidation of cyclooctane using Mn(TMPyP) immobilized on MCM-41 (**A**) and SBA-15 (**B**); conditions: 60 mL of cyclooctene, cyclooctene:O_2_ ratio equal to 6.5, catalyst loading adjusted to 0.001 g of chloromanganese(III) 5,10,15,20-tetrakis(4-*N*-methylopyridynio)porphiryn tetrachloride Mn(TMPyP) [51].

Chloromanganese(III) 5,10,15,20-tetrakis(4-N-methylopyridynio)porphiryn tetrachloride Mn(TMPyP) was immobilized on MCM-41 and SBA-15, both materials modified by alumination [51]. Used supports were aluminated by both direct and post-synthesis method. In direct method, aluminum was introduced during the mesoporous material synthesis, while in post-synthesis method purely siliceous materials were modified. The Mn-porphyrin has been encapsulated into aluminated mesoporous silicas by means of cationic exchange. The catalysts were tested in the oxidation of cyclooctane with molecular oxygen (10 atm, 120 °C), and the conversion rates and cyclooctanone/cyclooctanol selectivities were determined. All studied mesoporous materials showed higher catalytic activity than unsupported porphyrin. Interestingly, dependence between pore size and conversion rate was observed, for SBA-15 modified directly with aluminum it was as high as 11.8%. However, the cyclooctanone selectivity was the highest (97–98%) for the samples of MCM-41 modified post-synthetically with aluminum (Scheme 1). This result was attributed to the small size of the MCM-41 mesopores, which may prevent escape of free radicals from the solvent cage, thus leading to ketone formation [51].

**Scheme 2:**
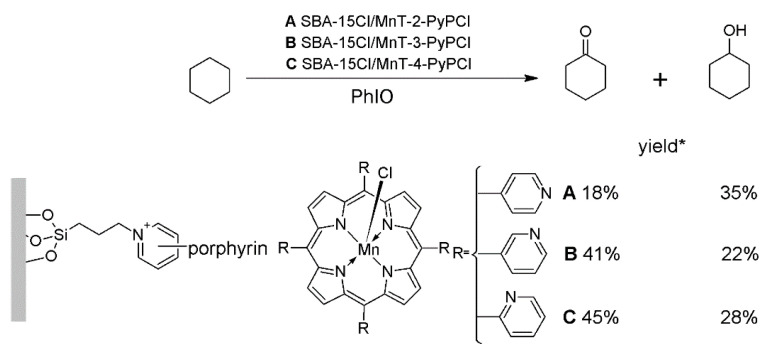
Oxidation of cyclohexane using three isomers of N-pyridylporphyrins on SBA-15: *para-* (**A**), *meta-* (**B**) and *ortho-* (**C**); MnP/PhIO/cylohexane molar ratios were of 1:10:5000, MeCN:CHCl_3_ (1:1, *v*/*v*), magnetic stirring under air, 25 °C, 90 min; * yields calculated based on PhIO [52].

Pinto et al. [52] chose SBA-15 mesoporous silica as a support, which is hexagonally ordered like MCM-41 but with larger pore size in the range of 5 to 15 nm [26,53]. Large pore-size mesoporous materials are desirable when it comes to immobilization and carrying out the reactions involving large molecules, such as porphyrins, that can be modified with substituents of various sizes. Above mentioned researchers used for immobilization three isomers of the neutral N-pyridylporphyrins (MnT-2-PyPCl, MnT-3-PyPCl and MnT-4-PyPCl) and three isomers of cationic N-methylpyridylporphyrins (MnTM-2-PyPCl_5_, MnTM-3-PyPCl_5_ andMnTM-4-PyPCl_5_). Solid catalysts were prepared by encapsulation of porphyrins using two methods based on: (1) covalent bonding of neutral porphyrin to chloropropyl-functionalized SBA-15Cl and (2) electrostatic interaction between cationic porphyrin and SBA-15. Immobilization of the neutral porphyrin on modified SBA-15 made it possible to obtain a more stable complex not susceptible to leaching. However, the functionalization is not directional, and the introduced chloropropyl groups may be on the surface as well as inside the mesopores and micropores of SBA-15, making it difficult to determine what amount of porphyrin has been encapsulated. Cyclohexane and iodosylbenzene as oxidant were selected for the catalytic reaction involving the above-mentioned complexes. For three isomers of neutral N-pyridylporphyrins on SBA-15, an increase in the yield of products was observed in the following order: para > meta > ortho (54%, 62% and 72% total yield) with the selectivity towards the alcohol (Scheme 2). Interestingly, comparing with the results obtained for not encapsulated porphyrin catalyst the trend is opposite and there were similar yields of alcohol and ketone for all isomers. These results show that the deposition on solid support can affect the selectivity of the products, in this case selectivity towards the alcohol–cyclohexanol. It was also noted that after reuse of the catalyst the more efficient complex turned to be cationic N-methylpyridylporphyrins, although it was less effective after the first reaction cycle [52].

**Scheme 3:**
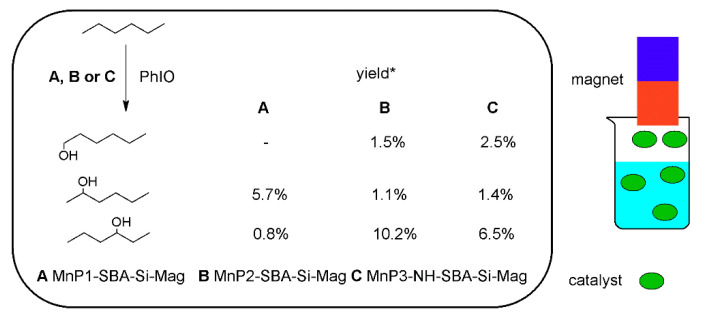
Oxidation of n-hexane using porphyins: MnP1-SBA-Si-Mag, MnP2-SBA-Si-Mag and MnP3-NH-SBA-Si-Mag modified with magnetite and their removing from reaction medium. MnP/PhIO/hexane molar ratios were of 1:50:5000, MeCN:CH_2_Cl_2_ (1:1, *v*/*v*), magnetic stirring under argon, 25 °C, 1h; * yields calculated based on PhIO [54].

The same researcher group took it a step further and modified SBA-15 with magnetite [54]. Such modification makes it easier to reuse the catalyst by removing from the solution with the aid of a magnet. As in the previous study, the researchers immobilized neutral porphyrin (MnP3—pentafluorophenylporphyrin) on APTES-functionalized SBA-15 (NH_2_-SBA-Si-Mag) and cationic (MnP1—4-N-methylpyridylporphyrin, MnP2—4-N-methylpyridyl) porphyrins in SBA-15 by covalent and electrostatic bonding, respectively. Prepared catalysts were used for the oxidation of cyclic alkane–cyclohexane, linear—n-hexane and adamantane as the representative of secondary and tertiary C-H bonds. The choice of the substrates made it possible to study the selectivity of the proposed catalysts in relations to the formation of ketone or alcohol and its ability to carry out the reaction in a regioselective manner, i.e., oxidation at a specific position in the molecule. During the oxidation of above-mentioned alkanes in the presence of PhIO, the selectivity towards the formation of alcohols was observed, while ketone showed up in small amounts as a product. During the oxidation of n-hexane, it was observed that only the cationic porphyrin MnP2 was selective for 1-carbon leading to the formation of 1-hexanol, which is not a favorite due to its high binding energy (Scheme 3). This was not observed in the case of reaction carried out by homogenous catalyst MnP2 without support. It proves that the immobilization on the mesoporous solid material influenced the selectivity of the conducted reaction. This may be the first time that the selectivity for oxidation of terminal carbon occurs in the reaction carried out by a supported metalloporphyrin. Nevertheless, the best results in the preparation of 2- and 3-hexanol (17.5% total yield) were obtained for the neutral porphyrin MnP3, and even better for the non-immobilized compound. Similarly, in the case of cyclohexane and adamantine oxidation, the best results were obtained for MnP3 (38.4% cyclohexanol yield and 28% 1-adamantanol yield). In every reaction the unsupported MnP3 compound showed similar or higher activity in oxidation than the immobilized one, suggesting that the best results obtained for MnP3 may be due to the deposition of porphyrin on the surface of SBA-15.

#### 3.1.2. Phthalocyanine-Base Catalysts

Although there are many examples of porphyrins used in the oxidation reactions of alkanes, phthalocyanines also attracted attention. As non-naturally occurring porphyrin analogues they also can be chosen to carry hydrogenation reactions.

Armengol et al. have been one of the first to immobilize MPcs on mesoporous materials to check their catalytic activity for the oxidation of cyclohexane. The Pcs complexes with MCM-41 and Y faujasite, a zeolite, were studied. Copper(II) phthalocyanine (CuPc) and cobalt(II) perfluorophthalocyanine (CoPcF_16_) were placed inside the pores of both materials using the ship-in-a-bottle method—through synthesis from 1,2-dicyanobenzene. The results obtained from differential scanning calorimetry indicated that MPc complexes are more stable when grafted onto MCM-41 than Y zeolite. The proposed interpretation is that it is related to different conformations: planar for MCM-41 and distorted for Y zeolite, which is directed by the pore size of the host material. The catalytic activities of the prepared complexes (50 mg, containing MPc equivalent to 0.53 mg of unsupported CuPc or CoPcF_16_) were tested in oxidation of cyclohexane (700 mg), using H_2_O_2_ (98 mg, 30%) or TBHP (200 mg) as oxidants; reaction medium was acetonitrile (7 mL). The main products were cyclohexanol and cyclohexanone, highest alcohol:ketone ratio (63:37) was observed for Y faujasite-bound CuPc and MCM-41-bound CoPcF_16_.TBHP was found to be a better oxidizing agent because did not lead to degradation of metallophthalocyanine complexes [55].

After the publication of results obtained by Armengol and co-workers many other researchers investigated the catalytic activity of metallophthalocyanines incorporated in different types of mesoporous materials. MCM-41 was chosen for the incorporation reaction more often than other types of mesoporous silicas.

**Scheme 4:**
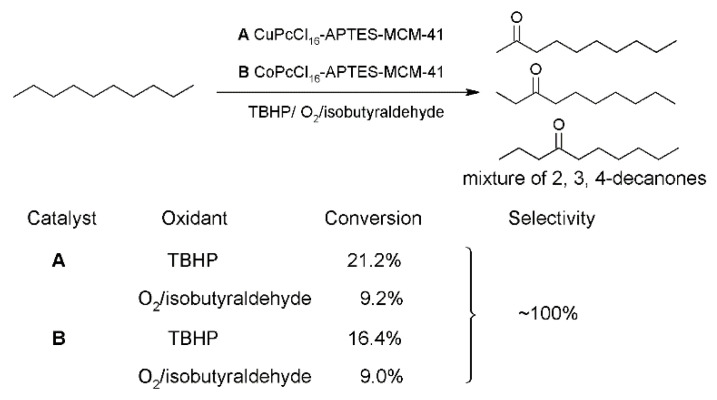
Oxidation of n-decane using Cu/Co-Cl_16_Pc immobilized on MCM-41 [56].

Copper(II) and cobalt(II) hexadecachlorophthalocyanines (CuPcCl_16_ and CoPcCl_16_, respectively) immobilized on APTES-functionalized MCM-41 were used for liquid phase oxidation of cyclohexane and *n*-decane (Scheme 4). These complexes had better catalytic activity and exhibited higher rates of reaction than Pcs grafted onto amino-functionalized SiO_2_ or on non-functionalized MCM-41. Authors tested two different oxidants: TBHP and O_2_/aldehyde in the oxidation reaction of cyclohexane and *n*-decane. The CuPcCl_16_ grafted on APTES-functionalized MCM-41 showed the highest conversion of decane in the presence of TBHP, was twice more active than O_2_/isobutyraldehyde [56].

In the aforementioned studies by Ernst and Selle ruthenium perfluorophthalocyanine incorporated into MCM-41 was used in the *n*-hexane and cyclohexane oxidation reactions [37]. TBHP (24 mmol) was used as an oxygen donor for oxidation of n-hexane (24 mmol) or cyclohexane (24 mmol) in acetone (20 mL) in the presence of 0.3 g of solid catalyst. In these studies, 2- and 3-hexanone were obtained as products in the oxidation of n-hexane. The results were completely different than in the case of the reaction carried out with the participation of porphyrins in the studies by Ucoski et al., where the alcohol derivatives were obtained [54]. Likewise, selectivity against position 1 and presence of 1-hexanol was also not observed. Researchers claims that the presence of ketones is due to the conversion of hexanol to hexanone during the course of the reaction. It was also observed that the ketone derivative—cyclohexanone, was obtained with higher yield than cyclohexanol in the reaction.

### 3.2. Oxidation of Alkenes

Basing on the number of reports on the oxidation of alkenes with the use of porphyrins and phthalocyanines deposited on mesoporous materials, it can be concluded that this is a field of research of great interest. This is due to the constant need to improve the methods of obtaining compounds useful in industry, i.e., products of cyclohexene oxidation. For example, cyclohexanol and cyclohexanone are necessary for the production of adipic acid, which is used in the production of nylon-6,6, used carpet fibers, upholstery, tire reinforcements, apparel and other products [57].

#### 3.2.1. Porphyrin-Based Catalysts

**Scheme 5:**
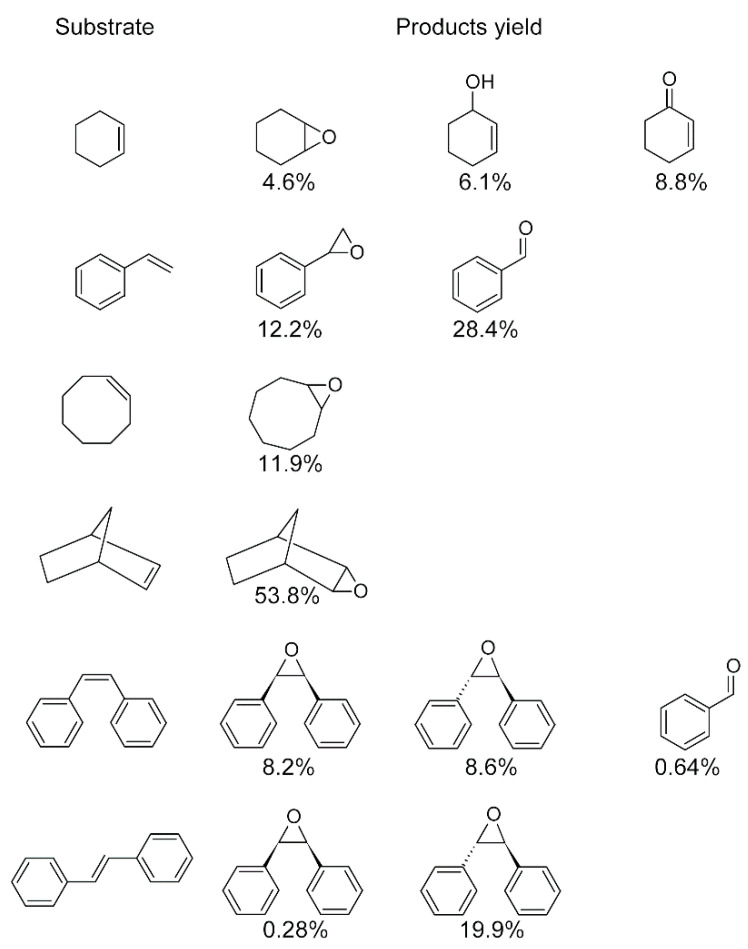
Oxidation products of alkenes using ruthenium(II) meso-tetrakis(4-chlorophenyl)porphyrin supported on MCM-41 [36].

Previously mentioned Liu et al. [36] used ruthenium(II) 5,10,15,20-tetrakis(4-chlorophenyl)porphyrin grafted onto APTES-modified MCM-41 complex for alkene oxidation using *tert*-butyl hydrogen peroxide. Experiments were conducted in a sealed vial under the nitrogen; alkene (0.2 g), TBHP (0.1 g), catalyst (0.05 g) The substrates for the oxidation reactions included norbornene, cyclohexene, cyclooctene, styrene, *cis*-stilbene and *trans*-stilbene (Scheme 5).

Cationic porphyrin—[meso-tetrakis(1-methyl-4-pirydinio)porphyrinato]manganese (III) penta-acetate was anchored to MCM-41 and NaY zeolite. Porphyrin loading was achieved by stirring manganese porphyrin with mesoporous silica and a base. The encapsulated catalysts exhibited a good catalytic activity, selectivity and stability in the epoxidation of styrene and cyclohexene by iodosylbenzene. The reaction conditions were as follows: iodosylbenzene 0.1 mmol, substrates (0.2 mmol), catalyst (0.005 mmol) were stirred in acetonitrile:dichloromethane (3:1) mixture (2 mL). Epoxide formation yields, based on iodosylbenzene consumption, were up to 51% and 91% for styrene and cyclohexene, respectively [58].

**Scheme 6:**
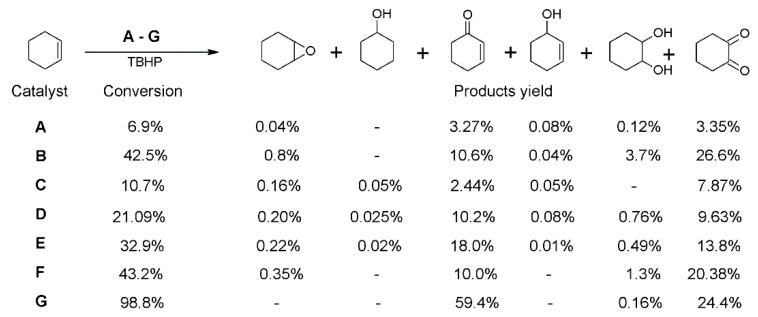
Oxidation of cyclohexene using series of porphyrins immobilized on MCM-41. (**A**): 5,10,15,20-tetraphenyl porphyrin; (**B**): 5,10,15,20-tetrapyridyl porphyrin; (**C**) 5,10,15,20-tetraphenyl porphyrin synthesized from pyrrole and benzaldehyde via “ship-in-a-bottle” method; (**D**) 3-aminopropyltrimethoxysilane-modified MCM-41 loaded with 5,10,15,20-tetrakis(pentafluorophenyl) porphyrin iron(III) chloride; (**E**) 5,10,15,20-tetraphenylporphyrin iron(III) chloride; (**F**) 5,10,15,20-tetrakis(pentafluorophenyl)porphyrin iron chloride; (**G**) 3-aminopropyltrimethoxysilane-modified MCM-41 loaded with 5,10,15,20-tetraphenylporphyrin iron(III) chloride [46].

Radha Rani tested aforementioned methods for effective encapsulation of iron(III) porphyrins in MCM-41 and NaY zeolite [46]. The reaction testing the catalytic activity was the oxidation of cyclohexene, using *tert*-butyl hydroperoxide as an oxidant (Scheme 6). The best results—highest conversion of cyclohexene and selectivity of cyclohexanone formation—were found for iron(III) 5,10,15,20-tetrakis(pentlafluorophenyl)porphyrin when anchored on Al-MCM-41 functionalized with 3-aminopropyltrimethoxysilane (APTMS). The catalyst remained active during four catalytic runs. Despite detailed description of the results, Authors do not provide reliable procedure of the catalytic reaction.

Efficiency of these materials used as active catalysts was studied by Costa and co-workers [59]. They applied Fe, Mn and Co complexes of 5,10,15,20-tetraphenylporphyrin (FeTPPCl, MnTPPCl and CoTPP, respecitvely) immobilized on MCM-41. The catalyst materials were obtained by a simple adsorption method, through stirring metalloporphyrin with MCM-41 in dichloromethane for 48 h. Such direct encapsulation of metalloporphyrins is possible because MCM-41 silicas have pore sizes larger than 20 Å. Different amounts of porphyrins were incorporated, which can be connected with the interaction of each complex with the MCM-41. The reaction studied was cyclohexene oxidation with hydrogen peroxide; which resulted in formation of cyclohexanol, cyclohexanone and cyclohexene oxide. Cyclohexene (10 mmol), H_2_O_2_ (12 mmol) and 0.1 g of the catalyst were stirred in acetonitrile (3 mL) under reflux for 8 h. According to the results, FeTPPCl/MCM-41 has higher catalytic activity than CoTPPCl/MCM-41 and MnTPPCl/MCM-41. Due to lower loading, MnTPPCl/MCM-41 showed lower activity, but the highest TON. Noticeable differences in the activity of complexes with different metal ions show that the catalytic activity largely depends on the metal ion in the coordination center (Table 1).

**Figure 4:**
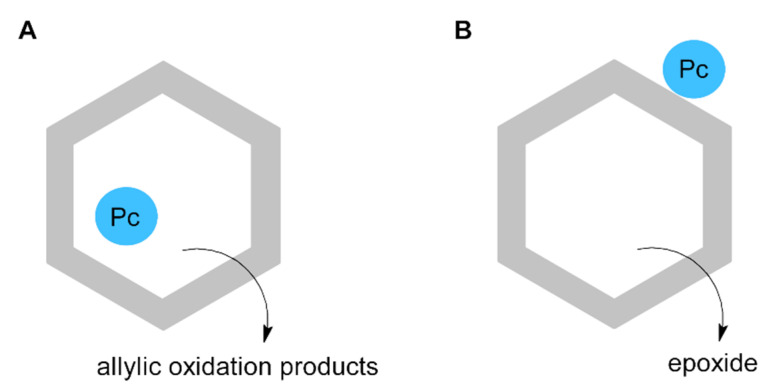
Direction of the oxidation reactions depending on the porphyrin location: inside the pores of support (**A**) and deposited on the external surface (**B**) [60].

It was also observed that despite the slight differences in activity, all metalloporphyrin complexes showed selectivity towards the allylic oxidation products over the epoxide derivatives. According to Zimowska’s concept, this may be due to the location place of porphyrins on the support [60]. Porphyrins anchored inside the pores of MCM-41 gave cyclohexanone and cyclohexanol as products of oxidation, while those deposited on the external surface were more selective towards the epoxide (Figure 4). It turns out that at the incorporation stage, we can predict the location of macrocycles on the support, as well as the direction of the oxidation reaction.

Chloromanganese(III) 5,10,15,20-tetrakis(4-N-methylopyridynio)-porphiryn tetrachloride (Mn(TMPyP)) was incorporated in aluminated FSM-16 by cationic exchange. The method of alumination determines where the porphyrin complex is accumulated on the support. Impregnation of mesoporous silica with aluminum results in the deposition on the surface, whereas direct alumination leads to incorporation inside the pore system. Cyclohexene oxidation using PhIO as oxidant and Mn(TMPyP) (800:20:1 molar ratio, porphyrin concentration was 0.67 mmol/L) was carried out in CH_2_Cl_2_:CH_3_OH (1:2) mixture (3 mL) for 16 h. Externally attached catalyst are not sterically hindered and an epoxide is formed. On the other hand, porphyrins encapsulated on direct aluminated FSM-16 favored allylic oxidation—up to 94% of products were cyclohexenol and cyclohexenon, while for the unsupported Mn(TMPyP) 86% of the product was cyclohexene epoxide. The method of mesoporous silica functionalization turns out to be a very important factor when it comes to the selectivity of product formation.

Rahiman and co-workers also decided to functionalize mesoporous material—MCM-41, but with titanium of different Si/Ti ratios [61]. The low Si/Ti ratio resulted in increased intake of porphyrin complex. Moreover, the strong binding of chloromanganese(III) 5,10,15,20-tetrakis(4-trimethylammoniophenyl)porphyrin chloride to titanium-substituted MCM-41 prevented leaching of the catalyst during the reaction. The cationic manganese porphyrin incorporated in such material by cationic exchange, was used for the oxidation reaction of cyclohexene and styrene—alkene (5 mmol), PhIO (0.5 mmol) and catalyst (0.025 mmol) in acetonitrile (6 mL) were stirred for 24 h under inert atmosphere Epoxides were obtained with selectivities up to 85% and 94% for cyclohexene and styrene, respectively, for MCM-41-bound porphyrin Introduction of titanium to MCM-41 resulted in lower selectivity, but increased yields.

A few years later, the same researchers continued their work using cationic porphyrin with oxovanadium(IV) instead of manganese in central cavity (oxovanadium(IV) 5,10,15,20-tetrakis(4-trimethylammoniophenyl)porphyrina tetrachloride; (VOTAPP)) anchored to mesoporous silica by electrostatic interaction [62]. The support used was MCM-41 with introduced vanadium and aluminum ions, respectively. The prepared complex of supported-porphyrin was subjected to oxidation of cyclohexene and styrene in the presence of PhIO, using above listed proportions. Not incorporated porphyrin as well as supported on Si-MCM-41 produces epoxides as the major products. For VOTAPP on Al-MCM-41, the selectivity of the products was opposite, where major components were allylic oxidation products. The distribution of products in the case of porphyrin supported on V-MCM-41 was completely different. In this case oxidation of cyclohexene led to 2-cyclohexene-1-one and 2-cyclohexene-1-ol, while oxidation of styrene produces epoxide. Higher epoxide yield in the case of using complex with V-MCM-41 can be explained by the Brønsted–Lowry theory. The exchange of Si for Al resulted in the formation of more acid sites which may cause opening of the epoxide ring. As the consequence the reduced yield of epoxide is observed in the reaction carried out by porphyrin incorporated in Al-MCM-41 (Table 2). As can be seen, the encapsulation of porphyrin on the support was of key importance as far as the selectivity of the reaction is concerned.

As in the case of the oxidation of alkanes by porphyrin catalysts, also here there are examples of modifying the mesoporous material with magnetite. It makes it possible to easily separate the catalyst from the reaction products in solution and allows the structure of the silica to be changed e.g., by tuning the pore size.

**Scheme 7:**
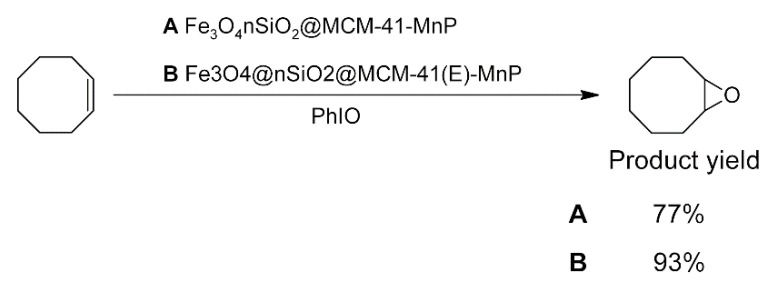
Oxidation of cyclooctene using Fe_3_O_4_@nSiO_2_@MCM-41-MnP and Fe_3_O_4_@nSiO_2_@MCM-41-(E)-MnP with larger pore size [63].

Magnetic Fe_3_O_4_ nanoparticle covered with SiO_2_ were obtained and then used in the synthesis of MCM-41 material [63]. Two materials with different pore size were obtained that way, and were further modified with APTES and loaded with manganese(III) 5,10,15,20-tetrakis(pentafluorophenyl)porphyrin. These materials were used as catalysts in oxidation of (Z)-cyclooctene and cyclohexane, using iodosylbenzene as an oxidizing agent (Scheme 7). Ratio of substrate:PhIO:porphyrin was 6140:100:1. Both catalysts were selective for cyclohexanol. Inorganic supports and solvent create a microenvironment which is suitable to carrying out catalytic reactions similar to P450-type biomimetic ones. Material with larger pores had a better catalytic efficiency for the alcohol product than the other one. Similarly, in cyclohexane oxidation, cyclohexanol was the favored product of the solid-state supported catalysts, while oxidations with homogenous porphyrin catalysts resulted in higher conversion of cyclohexane, but also in mixture of products—cyclohexanol and cyclohexanone. Moreover, the reusability tests were carried out, the expended-pores catalysts was once again superior, as it was stable for eight cycles, compared to five cycles for non-expanded [63].

**Scheme 8:**
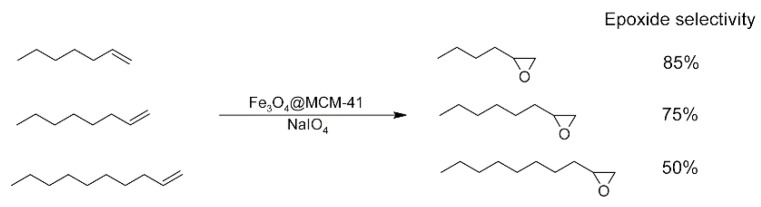
Oxidation of linear alkenes using Fe_3_O_4_@MCM-41 porphyrin.

The chloromanganese(III) 5,10,15,20-tetraphenylporphyrin (MnTPPCl) is another example of deposition on a magnetite modified support [64]. Mesoporous silica MCM-41 was also functionalized with 3-chloropropyltriethoxysilane (CPTES) and imidazole, which allowed the axial ligation of porphyrin to mesoporous material. Prepared complex was used in the oxidation of numerous of cyclic and linear alkenes: cyclooctene, cyclohexene, styrene, α-methylstyrene, α-pinene, limonene, 1-hexene, 1-octene and 1-decene, with NaIO_4_ as oxidant. In typical experiment alkene (0.5 mmol), Fe_3_O_4_@MCM-41-Im@MnTPPCl (200 mg) and NaIO_4_ (1 mmol) were stirred in acetonitrile:water mixture (8 mL, 5:3 *v*/*v*). Conversion of the alkenes were up to 98% (for cyclooctene). In all reactions the epoxide was the main product with the highest yield. It was observed that in the case of linear alkenes oxidation, the epoxide yield decreased with the chain length (Scheme 8). This may be due to the reduced availability to the active site of the catalyst. The proposed combination of porphyrin with Fe_3_O_4_@MCM-41-Im support, showed good reusability without decrease in activity during catalytic cycle.

In yet another study, three complexes derived from 5,10,15,20-tetrakis(4-chlorphenyl)porphyrin (TClPP) were used and immobilized in MCM-48 silica channel [65]. MCM-48 materials containing manganese(III), iron(III) and cobalt(II) complexes of this porphyrin were employed as catalysts for cyclohexene oxidation with TBHP as the oxidant. Since MCM-48 has three-dimensional channels which provide easy access of the reactants to the active sites of the metalloporphyrins, it is expected to provide better diffusion and resistance to pore blocking, than MCM-41, which has unidirectional pore system. The results obtained from inductively coupled plasma (ICP) spectroscopy showed that porphyrin content was up to 17.6%, for FeTClPP. The catalytic activity of the complexes of FeTClPP-MCM-48 with different amounts of Fe(III) porphyrin was tested. In typical experiment, mixture of cyclohexene (10 mmol), TBHP (15 mmol) and 0.02 g of heterogeneous catalyst were stirred in acetonitrile (5 mL) for 6 h. The results demonstrated that an increase in the amounts of metalloporphyrin loading led to an increase in catalytic activity. This was attributed to the three-dimensional structure of MCM-48, because in case of MCM-41 an increase in catalyst loading frequently led to decrease in activity, due to blocking of the pores. Furthermore, increase in the reaction temperature improved the conversion of cyclohexene to 2-cyclohexene-1-one as the main product. The Fe(III)TClPP-MCM-48 showed the best catalytic activity, better than both MnTClPP-MCM-48 and CoTClPP-MCM-48 in the oxidation of cyclohexene (Table 3). The results obtained by Khalili indicated that the support played an important role and had a significant effect on the catalytic efficiencies [65].

Najafian et al. have investigated oxidation of cyclohexene using copper(II) 5,10,15,20-tetrakis(4-chlorophenyl)porphyrin (CuTClPP) immobilized onto SBA-16 modified by 3-aminopropylthetoxysilane. It was performed, using the aforementioned “ship-in-a-bottle” method (Figure 3c); the porphyrin was synthesized by reacting copper(II) acetate, 4-chlorobenzaldehyde and pyrrole. The catalytic activity of the catalyst was examined in the presence of TBHP and hydrogen peroxide as oxidants. Experiments mixture contained cyclohexene (10 mmol), oxidant (10 mmol), acetonitrile (5 mL) and various amounts of the catalyst and were stirred at room temperature for 3 h, under in dark or under visible light irradiation. Light source was a 100 W cannular tungsten lamp. The results showed that the activity of the catalyst increased when exposed to visible light. The obtained mixture (remaining cyclohexene and the oxidized products) were characterized by gas chromatography (GC). The blank experiment with pure support (SBA-16) and NH_2_-SBA-16 did not show any oxidation products which indicates that metalloporphyrin complexes encapsulated in SBA-16 play the main role in the catalytic activity. In optimal conditions (TBHP as an oxidant and under light irradiation), cyclohexene conversion was 76%, and selectivity towards cyclohexenone was 93% [44].

A complex of manganese(III) 5,10,15,20-tetraphenylporphyrin acetate (MnTAPP) immobilized on MCM-48 was investigated in its catalytic activity in cyclohexene oxidation [39]. The aim of the study was also to check the effect of nanoporous structure on the catalytic efficiency by comparing the results with those obtained for colloidal silica (SiO_2_). Previously described pyridine linker was used to immobilize MnTAPP inside MCM-48 (Figure 2b). Urea hydrogen peroxide (0.1 mmol) used as an oxidizing agent, cyclohexene (0.05 mmol) and MCM-48-bound porphyrin (0.1 g of the material) were stirred in various solvents: methanol, acetonitrile, tetrahydrofuran and dichloromethene. The results showed that the best solvent for cyclohexene oxidation was methanol, as process conducted in this solvent showed highest conversion (>95%) and high yield of cyclohexene oxide (~85%). Authors rationalize that methanol could act as an acid catalyst accelerating heterolytic cleavage of the O-O bonds in the peroxide species, which leads to the formation of active form of the catalyst. In order to investigate the effect of nanoporous structure on the catalytic efficiency, MnTAPP was immobilized also on pyridine-functionalized colloidal silica. The results were better for the complex immobilized on MCM-48 than for that on colloidal silica, which can be attributed to the nanoporous structure of the former. It means that the porous structure increases the surface area and improves the catalytic activity of the catalyst [39].

**Scheme 9:**
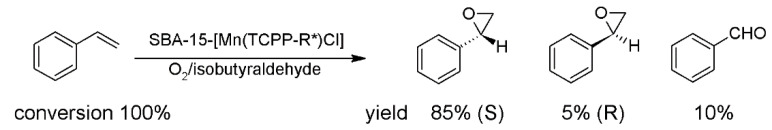
Enantioselective oxidation of styrene using chiral manganese(II) porphyrin; styrene (5 mmol), isobutylaldehyde (5 mmol) and mesoporous material (10 mg) were stirred in acetonitrile (5 mL) in oxygen atmosphere at 45 °C for 8 h [66].

Further examples relate to the catalytic performance of porphyrins incorporated into SBA-15 and SBA-16 mesoporous material type. Manganese(III) porphyrin, bearing a tartrate moiety as a chiral center, covalently bonded with SBA-15 (SBA15-[Mn(TCPP-R*)Cl]) was prepared and studied its chiral recognition in olefins oxidation reaction, using O_2_/isobutyraldehyde as oxidant [66]. The obtained results indicate the enantioselectivity of studied composite SBA15-[Mn(TCPP-R*)Cl]. During the oxidation of styrene 90% epoxide selectivity were obtained with 89% enantiomeric excess (Scheme 9). The obtained values were higher than those of α- and β-methylstyrene. However, in the oxidation of α-methylstyrene the enantiomeric excess (81%) was higher than that of unsupported porphyrin catalyst (Mn(TCPP-R*)Cl) (70%). It suggests that mesoporous support has an effect on asymmetric catalysis. Moreover, it can be concluded that the use of chiral catalyst allows chirality to be introduced into the final product.

Another chiral catalyst designed to achieve enantioselectivity. was manganese-tetrapyridylporphyrin (Mn(TPyP)) anchored to CPTMS-modified SBA-15 by covalent interaction and replaced its chloride and acetate ions by L-tartrate anion [67]. Such an exchange resulted in the appearance of chirality, which resemble the active site in enzymes belonging to the cytochrome P-450 family. Complex of SBA15-[Mn(TPyP)TA] was used in the oxidation of numerous linear, cyclic and aromatic olefins in the presence of O_2_. In the oxidation of styrene the conversion (100%) and epoxide selectivity (86%) were remarkably higher than the results obtained for catalyst without tartrate ion (93% and 74%, respectively). This example shows that tartrate ion causes the introduction of chirality to the products and remarkably affects the activity and selectivity in oxidation reactions. Moreover, there was no evidence of enantioselectivity when non-immobilized porphyrin carried out oxidation of styrene. This confirms that tartrate ligand is a key factor in inducing chirality and appearance of stereogenic center in the product.

Catalytic activity of vanadyl 5,10,15,20-tetrakis-(4-methoxycarboxyphenyl)porphyrin covalently-linked to APTES-functionalized SBA-15 [68] was also tested. Vanadium was selected due to its multiple oxidation states and high oxidation potential. Complex VOTMCPP@ N-SBA-15 was used for oxidation of cyclohexene at room temperature with hydrogen peroxide as oxidant. The reaction led to allylic products achieved with the highest yield (2-cyclohexene-1-one) with the conversion of 85%. In comparison with the previously reported catalysts with Co, Fe and Mn ions (maximum conversion 25.8%), vanadyl porphyrin show high catalytic activity. It can be considered as good transition metal for oxidation reaction of cyclohexene.

**Figure 5:**
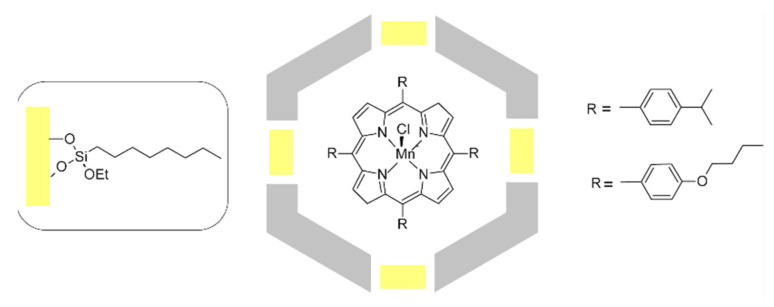
Schematic incorporation of two manganese porphyrins in SBA-15 modified with octyltriethoxysilane [69].

Another study aimed to solve the problem of porphyrin leakage from the mesoporous material during the catalytic reaction [69]. Two manganese porphyrins were encapsulated into SBA-16 i.e., chloromanganese(III) 5,10,15,20-tetrakis(4-isopropylphenyl) porphyrin (MnTIPP) and chloromanganese(III) 5,10,15,20-tetrakis(4-butoxyphenyl)porphyrin (MnTBPP). The encapsulation was followed by narrowing the pores by reacting the material with octyltriethoxysilane—a silylating agent with a long chain, to prevent leaching of porphyrin from the nanocages (Figure 5).

Afterwards, the prepared porphyrin catalysts (0.1 g, containing 0.0055 mmol and 0.0051 mmol of MnTIPP and MnTBPP, respectively) were subjected to oxidation reaction of cyclohexene, cyclooctene, 1-hexene and 1-octene (4 mmol), using sodium hypochlorite (20 mmol) as oxidant and imidazole (5 mmol) as co-catalyst in dichloromethane, in presence of tetrapropylammonium bromide (4 mmol), as phase-transfer agent. Both catalysts showed good catalytic activity with the selectivity towards the epoxide, conversions of cyclohexene and cyclooctene were nearly 100%. The change of the pore size did not prevent the flow of reactants inside the pores and products out of the nanocages. Moreover, it inhibited the escape of large porphyrin catalyst, as evidenced by leaching of porphyrin at the level of 6% after 5 cycles. Judging by the results, the pore blocking method appears to be effective in reducing catalyst efflux from the support.

Chloromanganese(III) 5,10,15,20-tetraphenylporphyrin (Mn(TPP)Cl) was immobilized onto three portions of SBA-15, functionalized with propyl-amine, -thiol and -sulfonic acid groups, respectively [70]. Such introduction of acid-basic properties of the support influenced the oxidation state, redox behavior and selective oxidation properties of the Mn porphyrins. The presence of acidic groups (-SO_3_H, -SH) promoted partial reduction of manganese ions in MnTPP to Mn(II) state. This reduction was confirmed by the EPR spectra, as Mn(II) porphyrin showed a hyperfine pattern, unlike Mn(III) complex, which is silent in EPR. Modified SBA-15 samples with built-in Mn(TPP)Cl were used as catalysts in oxidation of *R*-(+)-limonene with aerial oxygen. Conventional limonene oxidation is conducted with stoichiometric amounts of peracids, which is not eco-friendly. Moreover, limonene oxidation may lead to a variety of products, e.g., epoxides, carveol and carvone. In a typical reaction, 0.05 g of Mn(TPP)Cl, *R*-(+)-limonene (3.75 mmol), isobutyraldehyde (9 mmol, co-reagent), *N*-methylimidazole (1.7 mmol) were stirred in toluene (20 mL) under air flow (1 atm, 2 mL/min) in room temperature for 8 h. Endo-1,2-epoxide was the main product of the reactions catalyzed by Mn(TPP)Cl immobilized on the modified SBA-15, and the propylsulfonic-functionalized was the most active catalytically (62% conversion of substrate, selectivity towards epoxide 95%), while the propylamine-modified was the least active. This observation may support the hypothesis that the oxidation state of Mn ions have strong a impact on catalytic activity, as catalyst based on propylsulfonic-functionalized SBA-15 had highest level of Mn(II) and highest activity [70].

#### 3.2.2. Phthalocyanine-Based Catalysts

**Scheme 10:**
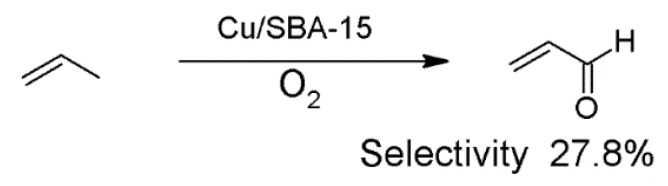
Oxidation of propylene using copper-modified SBA-15, copper(II) phthalocyanine was the source of copper [71].

Very specific usage of copper tetrakis(methylpyridinium)phthalocyanine tetrachloride (CuPc) immobilized onto SBA-15 was implemented [71]. Mesoporous material with CuPc was calcined which led to the oxidation of phthalocyanine. The complexes of CuPc with SBA-15 with different loadings were tested under different reaction conditions. From among the complexes tested the most active was the containing the smallest amount of copper (0.06 wt% Cu). The oxidation selectivity towards acrolein (Scheme 10) increased with decreasing loading of copper (11% at 0.9 wt% Cu and 42% at 0.06 wt% Cu). It can be related to the fact that copper remains in a highly dispersed state attributed to the low copper loading which prevents aggregation. It is assumed that isolated copper sites are responsible for the selective oxidation reaction. The same authors have tested the influence of different reaction conditions on the effectiveness of oxidation process. The highest activity and selectivity was obtained at 475–500 °C. Additionally, with increasing oxygen partial pressure the selectivity to acrolein decreased (from 54.8% to 28.1%) and conversion increased took place (from 6.7% to 30.5%).

Iron(II) tetrasulfophthalocyanine (FePcS) was immobilized in APTES-modified MCM-48 and MCM-41 [72]. The complex was used as a catalyst for oxidation of styrene with TBHP leading to benzaldehyde. Mesoporous materials used varied in structure MCM-41 with the two-dimensional hexagonal array of pore channels and MCM-48 with three-dimensional pore network and larger surface area. The advantageous features of MCM-48 make it more favorable for the catalytic reaction, but it is difficult to obtain the material with controllable pore size and high quality. Catalytic procedure was as follows: styrene (0.025 mmol), mesoporous material (22 mg, 4% loading) and TBHP (0.025 mmol, added in portions) were stirred in water/methanol mixture (5 mL, 1:1 *v*/*v*) for 6 h at room temperature. Homogenous catalysis served as a comparison. The conversions in both systems were comparable but the homogeneous one gave benzoic acid as an undesirable product. The results confirmed that FePcS immobilized on MCM-48 showed higher activity than when grafted onto MCM-41 (Table 4). It can be related to the three-dimensional different pore structure of MCM-48, and thus better dispersion into the pores as such a pore structure provides easier access of guest molecules [72].

### 3.3. Oxidation of Aromatic Compounds

#### 3.3.1. Porphyrin-Based Catalysts

**Scheme 11:**
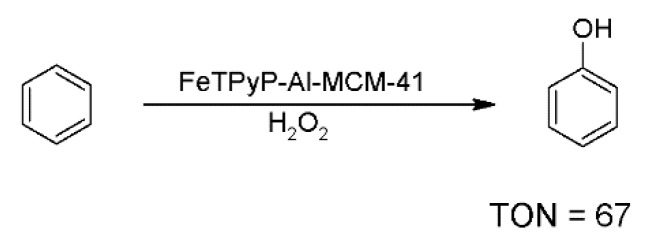
Oxidation of benzene using FeTPyP immobilized on Al-MCM-41 [73].

Oxidation of benzene to phenol was studied using iron(III) 5,10,15,20-tetrakis-(4-pirydyl)porphyrin as a catalyst and hydrogen peroxide as an oxidant [73] (Scheme 11). The catalyst was incorporated into Al-MCM-41 and poly(methacrylic acid) (PMAA), the authors have compared the effectiveness of the free and incorporated catalyst. Reaction procedure was as follows: benzene (2 mL), 30% aqueous H_2_O_2_ (1 mL), catalyst (50 mg) and methanol (2mL) were stirred at 70 °C. Interestingly, porphyrin used in free form showed no catalytic activity. The PMAA-bound porphyrin complex had higher catalytic activity than that immobilized on Al-MCM-41. However, molecular sieves-bound porphyrin showed higher selectivity, as phenol was the only product, while after oxidation using PMAA complex, hydroquinone was also observed. Additionally, Al-MCM-41-supported porphyrin showed better reusability; when materials were recovered and reused in next catalytic process, 100% and 75% of initial activity was found for Al-MCM-41 and PMAA materials, respectively. According to Nur et al. that the ordered structure of the complex with Al-MCM-41 may contribute to a high selectivity [73].

**Scheme 12:**
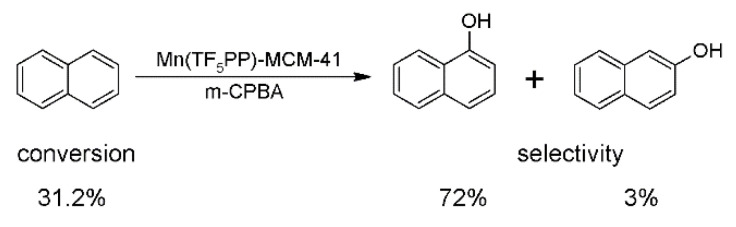
Oxidation of naphthalene using manganese porphyrin immobilized on MCM-41 [74].

MCM-41 modified with APTES porphyrins was loaded with different amounts of chloromanganese(III) 5,10,15,20-tetrakis-(pentafluorophenyl)porphyrin (TF_20_PPMnCl) [74]. The materials were used as catalysts in the oxidation reaction of naphthalene in the presence of *meta*-chloroperoxybenzoic acid (*m*-CPBA) as an oxidant. Naphthalene (1 g), *m*-CPBA (naphthalene/*m*-CPBA molar ratio = 1/1.2) catalyst (0.1 g) were stirred in acetonitrile/dichloromethane (40 mL, 1:1 *v*/*v*) at 30 °C for 4 h. Then the products were analyzed by gas chromatography. 1-Naphtol was the major product (ca. 70%) of the reaction, while 2-naphtol was the minor product (less than 5%) (Scheme 12). Materials with different loading of the catalyst were compared, an increase in the loading with manganese porphyrin led to an increase in the catalytic activity of the material. Compared with the homogenous porphyrin catalyst, modified MCM-41 showed lower activity (conversion 31.2% vs. 54.5%) and selectivity (no 2-naphtol was observed for homogenous catalysis). The lower activity of the modified MCM-41 was attributed to the occupancy of the one face of porphyrin by coordinated-NH_2_ group. The samples prepared showed good catalytic activity in the hydroxylation of naphthalene and remarkable reusability. These properties can be due to the oxidation of manganese porphyrins to manganese peroxy complexes which react with substituents at the naphthalene ring. In this reaction manganese porphyrin is involved in oxygen transferring [74].

#### 3.3.2. Phthalocyanine-Based Catalysts

**Scheme 13:**
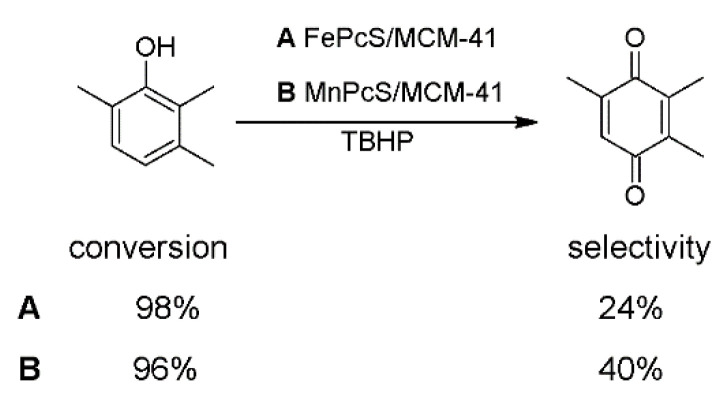
Oxidation of 2,3,6-trimethylphenol using iron and manganese phthalocyanines immobilized in MCM-41 [42].

Metallophthalocyanines linked covalently to MCM-41 were used as catalyst for selective oxidation of aromatic compounds, important for vitamin synthesis—2-methylnaphthalene and 2,3,6-trimethylphenol [42]. It is of great practical interest to develop such catalyst which would allow obtaining compound related to vitamin K and the intermediate of synthetic vitamin E (Scheme 13) without causing environmental problems. Three complexes of tetrasulfophthalocyanine were used; with various metal ions: manganese(II)—MnPcS, cobalt(II)—CoPcS and iron(III)—FePcS. They were converted to corresponding tetrasulfochlorophthalocyanine complexes by treatment with SOCl_2_ or PCl_5_, and covalently linked to APTES-modified MCM-41 (Figure 2e). FePcS was linked to mesoporous material in monomeric form, or as μ-oxo-dimer. Two other iron(III) Pcs: hexadecachlorophthalocyanine (FePcCl_16_) and tetraaminophthalocyanine (FePc(NH_2_)_4_) were synthesized and linked to APTES-modified and CPTMS-modified MCM-41 (Figure 2f), respectively. TBHP was used as an oxidant for oxidation of 2-methylnaphthalene, while products were desired 2-methylnaphthoquinone accompanied by 6-methylnaphthoquinone and 2-naphtoic acid. After 24 h of the catalytic reaction the most active complexes turned out to be FePcS@MCM-41 with dimeric form of FePcS and monomeric FePcS@MCM-4, while both CoPcS and MnPcS complexes show low catalytic activity. Despite relatively high conversion, up to 81%, reaction was not very selective; 2-methylnaphthoquinone:6-methylnaphthoquinone ratio was 3:1. A surprising outcome of the study was that the dimeric iron(III) tetrasulphophthalocyanine was more active and more selective than the monomeric form. Authors stipulate, that the reason for the better selectivity of the dimer supported catalyst can be that additional radical forms are not generated during the catalytic reaction. Their formation could lead to side reactions which would decrease the efficiency of oxidation. Catalytic oxidation of 2,3,6-trimethylphenol to trimethylquinone by TBHP gave higher yields when free form or SiO_2_-loaded FePcS were used, rather that FePcS@MCM-41.

Vanadyl tetraaminophthalocyanine (TAVOP) was used in hydroxylation reaction of benzene [75]. The synthesized phthalocyanine was incorporated into mesopores of SBA-15 functionalized with CPTMS, which resulted in its covalent bonding, similarly iron(III) analogue, presented in Figure 2f. The prepared complex TAVOPc@Cl-SBA was tested in benzene hydroxylation in the presence of O_2_, which led to phenol production. Reactions in various solvents were conducted, focusing on their nature and properties. The optimal conditions were as follows: benzene (1 mL), catalyst (20 mg) and acetonitrile/water (5 mL, 4:1 *v*/*v*) were stirred at 80 °C for 12 h, under oxygen atmosphere (10 bar). Such conditions resulted in 9.3% yield of phenol, while increasing oxygen pressure to 13 and 15 bar resulted in side-reaction and resorcinol formation. Other solvents tested, such as pure acetonitrile, acetone or acetic acid resulted in 6.0%, 5.3% and 4.5% yields, respectively. The results seem to be encouraging, as the very important industrial material, phenol, was obtained in single-step using oxygen as an oxidant.

**Scheme 14:**
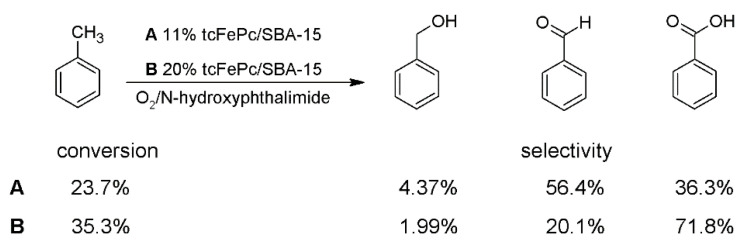
Oxidation of toluene using tcFePc with different content of phthalocyanine ((**A**)—11%, (**B**)–20%) incorporated in SBA-15; toluene (28 mmol), NHPI (2.0 mol%) and catalyst (0.03 g) stirred in acetonitrile (3 mL) in O_2_ atmosphere (balloon) [76].

In another study, APTES-functionalized SBA-15 was used for covalent immobilization of iron(II) tetracarboxyl-phthalocyanine (tcFePc) through amide bond formation [76]. Prepared catalyst was used for oxidation of toluene in the presence of molecular oxygen and N-hydroxyphthalimide (NHPI) as co-catalyst. The catalytic activity depended on the content of phthalocyanine and amino groups on mesoporous silica surface. The oxidation reaction led to benzaldehyde and benzoic acid as main products. It was noticed that the reaction conducted by phthalocyanine with a high degree of attachment to silica surface (with the surface area 188 m^2^/g) and the presence of less isolated amino groups (2.4 µmol/m^2^) led to the formation of benzoic acid (71.8% selectivity). Conversely, where complex had less grafted phthalocyanine and more isolated amino groups the main product of toluene oxidation reaction was benzaldehyde (56.4% selectivity) (Scheme 14). The conversion was 35.3% and 23.7%, respectively. These results shows that the catalytic properties depend on the phthalocyanine distribution inside the pores, as well as the pore volume and surface composition. This has a large impact on the diffusion of substrates and products inside and outside the pores of mesoporous support and determines the selectivity of the catalytic reaction.

### 3.4. Oxidation of Phenols

#### 3.4.1. Porphyrin-Based Catalysts

Application of SBA-15-supported iron(III) 5,10,15,20-tetrakis(4-pyridyl)-porphyrin (FeTPyP) as a catalyst for degradation of pentabromophenol was investigated using KHSO_5_ as an oxygen donor [77]. FeTPyP was covalently linked to the CPTMS-modified SBA-15. Bromophenols are brominated flame retardants widely used in production of electronic equipment, furniture or plastic. Bromophenols show endocrine disruption effects, and are found in surface water, soils and landfill leachates. Humic substances (HSs) accompany bromophenols in environmental samples, which affects the lifetimes of reactive oxidants and decreases the efficiency of the oxidative degradation of organic pollutants. SBA-15 was selected due to its small pore size which prevents the access of humic substances to the catalytic center in the channel of SBA-15. Thus, the inhibitory effects of HSs on the degradation reaction are suppressed and bromophenols can be selectively degraded. To test this hypothesis, FeTPyP immobilized on SiO_2_ was used as a control catalyst. The prepared FeTPyP-SBA-15 showed a high catalytic activity and 50 μM of pentabromophenol was efficiently degraded at pH 7 and 8, up to a concentration of 50 mg/L HS. As SiO_2_-based material showed significantly lower activity. Therefore, high selectivity of FeTPyP-SBA-15 was confirmed; well-ordered channels serving as a size selector, decreasing the blocking effect of humic substances in the efficacy of oxidative degradation [77].

#### 3.4.2. Phthalocyanine-Based Catalysts

**Scheme 15:**
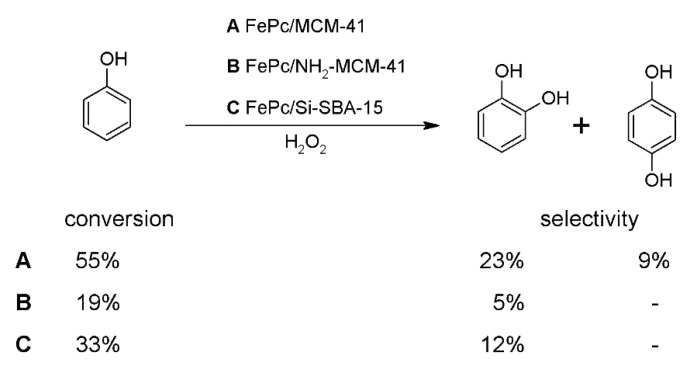
Oxidation of phenol using iron phthalocyanines incorporated in mesoporous silica using three methods: adsorption (**A**), coordination with NH_2_ groups (**B**) and covalent bonding (**C**); conditions: water (10 mL), phenol (1 g), catalyst (0.01 g) and H_2_O_2_ (2 mL, 25%) were stirred at 60 °C for 2 h [78].

Catalytic activity in phenol oxidation of iron(III)(acac)2,9(10),16(17),23(24)-tetra-tert-butylphthalocyanine deposited on the SBA-15 and MCM-41 carrier was also studied [78]. The phthalocyanine was immobilized using three methods: (i) physical adsorption on MCM-41, (ii) coordination with amino groups of MCM-41 surface and (iii) covalent bonding with silanol groups of SBA-15. The advantage of the third method is prevention of the phthalocyanine leakage. It turned out that such deposition protected phthalocyanine from oxidative degradation during the reaction, while other complexes had no catalytic activity after reuse. In phenol hydroxylation reaction with the use of H_2_O_2_ as oxidant, the complexes prepared by adsorption and covalent bonding was the most active catalyst with selectivity towards catechol (23% and 12%, respectively) (Scheme 15). The way phthalocyanine is incorporated into mesoporous material affects the arrangement of the molecule inside the pores. Increased access to unoccupied axial position of Fe(III) atom in phthalocyanine core center may have a significant effect on catalytic activity, as in the case of complexes prepared via (i) and (iii) method.

### 3.5. Other Uses

A series of tetracarboxylphthalocyanines was synthesized [41] The metallophthalocyanines were subsequently converted to acyl chlorides and covalently grafted onto MCM-41 functionalized with APTES (Figure 2d). The catalytic properties of the complexes were tested in oxidation of ethanethiol and thiophene as substrates, using oxygen as an oxidizing reagent. The oxidation reaction was strongly dependent on the reaction time. From all complexes studied, cobalt(II) tetracarboxylphthalocyanine with MCM-41 provided the highest conversion for both thiophene and ethanethiol, however little detail is given on the reaction conditions.

The efficacy of catalysis using cobalt tetrasulfophthalocyanine (CoTSPc) encapsulated in different mesoporous silicas (MCM-41, MCM-48 and SBA-15), zeolite (ZSM-5) and macroporous alumina (γ-Al_2_O_3_) was studied [79]. The materials were used to catalyze the oxidative degradation of azo dye C. I. Acid Red 73 in water using H_2_O_2_. In typical experiment 0.1 mg of dye, 30 mg of the catalyst and 10 mmol of H_2_O_2_ were stirred in 10 mL of buffered (pH = 7) solution. MCM-41 turned out to be the best support for CoTSPc, followed by SBA-15. The other materials showed little or no advantage, as compared to free-form CoTSPc. Catalytic potential of the CoTSPc@MCM-41 in oxidative degradation of other dyes (azo and antraquinone) was also showed. Authors stipulate that MCM-41 provides separation of CoTSPc aggregates into monomers by tightly anchoring the molecules inside the mesopores. Therefore, aggregation of the catalyst can be avoided, and the catalytic activity of CoTSPc for the degradation of dye pollutants is improved. The crucial factor for catalytical activity was the pH at the time of material preparation; as CoTSPc is monomeric above pH 10, materials prepared in these conditions were active in azo dye degradation. CoTSPc@MCM-41 prepared at lower pH (pH < 4) showed decreased catalytic activity [79].

## 4. Immobilized Porphyrins and Phthalocyanines as Catalyst in Photocatalytic Reactions

### 4.1. Porhyrin-Based Catalysts

Iron(III) 5,10,15,20-tetrakis(2,6-dichloro-4-sulfonylphenyl)porphyrin was covalently anchored to the surfaces of APTES-modified MCM-41 and SiO_2_. Oxygen atmosphere and irradiation with polychromatic light of wavelength range above 350 nm were applied. Oxidation of 1,4-pentanediol resulted in two products—4-hydroxypentanal and 5-hydroxy-2-pentanone, without producing ketoacids. The MCM-41-bound porphyrin showed a higher photocatalytic activity than that bound to the surface of silica. Additionally, mesoporous material with porphyrin showed preference towards production of 4-hydroxypentanal. The mesoporous catalyst has larger surface area; the iron porphyrin is better dispersed, thus provides a greater number of photocatalytic sites. It should be emphasized that iron porphyrin can be employed for the photocatalytic oxidation of 1,4-pentanediol only after its immobilization on MCM-41 or SiO_2_. The results indicated that surface properties of functional materials are crucial for developing a robust and efficient photocatalytic system [80].

An efficient photocatalyst was produced by immobilizing vanadium-doped mesoporous TiO_2_/5,10,15,20-tetrakis(4-carboxyphenyl)porphyrin (TiO_2_/TCPP) onto SBA-15. The materials synthesized was then investigated for degradation of 2,4-dichlorophenol (2,4-DCP) in aqueous solution and under visible light irradiation. TiO_2_ is a good metal oxide semiconductor which can generate highly oxidizing species, such as hydroxyl radicals in the photocatalytic process. One of its disadvantages is a low surface area—using mesoporous SBA-15 we can solve this problem and enhance the efficiency of a photocatalytic reaction. In this work SBA-15 was first pre-anchored with APTES and then functionalized with V-TiO_2_/TCPP. The vanadium doping increased the photoactivity of TiO_2_ acted as a hole (or electron) trap center while porphyrin acts as a light harvesting agent—producing photoinduced electrons and holes. Furthermore, TCPP can generate highly active oxygen: singlet oxygen or various reactive species such as superoxide radicals. The decomposition of 2,4-DCP starts after generation of singlet oxygen or superoxide radicals by porphyrin upon irradiation. The catalyst (25 mg) was stirred with 50 mL of solution containing 300 ppm of 2,4-DCP and was irradiated using a 400 W HQL lamp; within 150 min. the concentration was reduced by 99%. The results show that the V-TiO_2_/TCPP immobilized on SBA-15 exhibits higher catalytic activity than free TiO_2_/TCPP. It can be related to the coupling effect of adsorbed porphyrin and vanadium ions as well as SBA-15—increasing its absorption [81].

### 4.2. Phthalocyanine-Based Catalysts

A series of Al-MCM-41 materials with incorporated zinc(II) phthalocyanines was obtained [82]. Tetrasubstituted Pcs bearing nitro (ZnTNPc), phenyloxy (ZnTPhOPc) and dimethylaminoethyloxy (ZnTDMAEOPc) substituents were incorporated into mesoporous material through stirring of corresponding phthalonitrile derivative with zinc(II) acetate dihydrate, Al-MCM-41 and DMF at 130 °C, for 12 h. The tetraiodide salt of zinc(II) tetra(*N*,*N*,*N*-trimethylaminoethyloxy)phthalocyaninate, ZnTTMAEOPcI) was obtained through quaternization of the ZnTDMAEOPc with methyl iodide. Afterwards, three Al-MCM-41 materials (with ZnTNPc, ZnTPhOPc and ZnTTMAEOPcI) were irradiated in the presence of oxygen and fenamiphos or pentachlorophenol with polychromatic light of the wavelength range 320–460 nm. Solution (25 mL) of fenamiphos (10^−4^ mol/L) or pentachlorophenol (10^−5^ mol/L) was stirred with the catalyst containing 25 mg of the phthalocyanine, with access to air. Three Philips HPW125 mercury discharge lamps were used as a source of UV radiation. During this experiment, the concentration of the pesticide decreased following pseudo-first order kinetics. Presence of oxygen is essential for the photodegradation of the pesticides, and singlet oxygen is involved in this reaction. The most active complexes turned out to be ZnTNPc@Al-MCM-41 and ZnTTMAEOPcI@Al-MCM-41.

In another study, the photocatalytic activity of tetrasulfonated cobalt phthalocyanine (CoTSPc) immobilized onto MCM-41 was investigated [40]. Mode of the phthalocyanine binding was previously described (Figure 2c). Diffuse reflectance spectra were employed to prove that CoTSPc exists mainly in its monomeric state, which is the most catalytically active form. To evaluate the catalytic activity of the complex obtained the authors studied decomposition of 2,4-DCP. The tests for degradation 2,4-DCP were run: (i) in dark, (ii) under visible light and (iii) under UV-A irradiation. Additionally, influence of H_2_O_2_ on the catalytic activity was tested. Catalyst was ineffective in dark, even in presence of H_2_O_2_. UV-A irradiation of the catalyst resulted in significant decrease of 2,4-DCP concentration, while UV-A in presence of H_2_O_2_ was the most effective system in oxidation of 2,4-DCP The proposed mechanism was based on superoxide radicals, generated during irradiation, and their subsequent reaction with hydrogen peroxide, resulting in the formation of hydroxyl radicals. Main degradation products were oxalic, acetic and malonic acid as final products, while chlorocatechol and chlorobenzoquinone are proposed as intermediates of the oxidation process. The effectiveness of the catalyst reuse was also examined. For this purpose, the above-mentioned reaction was repeated in four cycle experiment. Recycling experiments showed the photocatalytic activity reduction—from 93% of 2,4-DCP removal in first experiment to 70% in fourth catalytic cycle. It can be attributed to deactivation of the part of the catalyst surface due to adsorption of intermediate species.

## 5. Summary and Outlook

Catalytic potential of porphyrins and phthalocyanines is a subject of a number of studies. To overcome typical difficulties such as recovery, reuse and macrocycle aggregation, the solid-state matrices were used. In this application, mesoporous silica is a promising candidate for anchoring tetrapyrrole macrocycles and obtaining new catalysts. Advantage of the heterogenic over homogenic catalysis is facilitated separation of the catalyst from the final product, and enable also using continuous-flow reactors [83]. Very interesting examples of mesoporous materials bound to magnetic particles were presented [54,63,64], this approach may further ease separation of the catalyst. Reproduction of processes carried by cytochrome P450 using catalysts in controlled conditions accelerates pharmacological and toxicological studies of many substances e.g., drugs and pesticides. Moreover, it enables synthesis of compounds which are difficult to synthesize chemically. Mesoporous materials-bound macrocycles are used primarily as catalysts in oxidation reactions. These approach meets various criteria of the green chemistry approach [84]. Using benign oxidants such as H_2_O_2_, oxygen or ideally air is a way to reduce need for oxidants based on the chromium(VI) or manganese(VII). Similarly, many of the reactions presented uses aqueous solutions as reaction medium. Additional “green” aspect of the described catalytic systems is their ability to oxidize environmental pollutants, including pesticides.

One of the challenges in developing efficient catalyst composed of porphyrin or phthalocyanine anchored to mesoporous silica is leaching of the macrocycle to reaction media. This may be prevented by anchoring the macrocycle molecule through coordination bonds [36,39] or covalent binding [40,41,42]. However, new approach by Masteri-Farahani et al. [69] (Figure 4) seems to have the advantage of locking the porphyrin molecule inside the mesopore while providing it enough freedom to be efficient catalyst. Another emerging challenge is the enantioselective synthesis [66,67].

We have demonstrated that the research on mesoporous structures and tetrapyrrole macrocycles opens the gates to their broad applications in different areas, such as the chemical and pharmaceutical industry, both in synthesis of basic industrial materials and fine chemicals.

## Figures and Tables

**Table 1:** Oxidation products of cyclohexene using porphyrins with different metals immobilized on MCM-41.

Catalyst	TON	%Conversion	%Selectivity		
			Epoxide	Cyclohexanol	Cyclohexenone
FeTPPCl/MCM-41	1.76 × 10^4^	25.8	25.9	46.0	28.1
MnTPPCl/MCM-41	1.56 × 10^5^	15.3	20.1	46.0	39.0
CoTPPCl/MCM-41	2.93 × 10^3^	11.8	23.9	33.9	37.1

**Table 2:** Catalytic performance of VOTAP porphyrin incorporated in Al-MCM-41 and V-MCM-41 in cyclohexene and styrene oxidation reaction.

Catalyst	Cyclohexene Conversion (%)	%Epoxide Yield
Al-MCM-41	43.7	3.3
V-MCM-41	47.8	5.5
	Styrene conversion (%)	%Epoxide yield
Al-MCM-41	20.3	5.7
V-MCM-41	45.3	28.2

**Table 3:** Oxidation of cyclohexene using FeTClPP, MnTClPP and CoTClPP porphyrins incorporated in MCM-48 [65].

Catalyst	TON	%Conversion	%Selectivity		
			Epoxide	Cyclohexanol	Cyclohexenone
FeTClPP-MCM-48	114.2	80	3.9	1.1	95.0
MnTClPP-MCM-48	144.6	65	11.4	3.4	85.2
CoTClPP-MCM-48	105.0	54	9.3	2.3	88.4

**Table 4:** Oxidation of styrene using iron phthalocyanine immobilized on MCM-41 and MCM-48 mesoporous support [72].

Catalyst	%Conversion	%Selectivity	
		Benzaldehyde	Benzoic acid
FePcS/NH_2_-MCM-41	46.9	20.2	-
FePcS/NH_2_-MCM-48	65.5	21.4	-

## Data Availability

Not applicable.

## References

[1] Panda M.K., Ladomenou K., Coutsolelos A.G. (2012). Porphyrins in Bio-Inspired Transformations: Light-Harvesting to Solar Cell. Coord. Chem. Rev..

[2] Collman J.P., Brauman J.I., Halbert T.R., Suslick K.S. (1976). Nature of O_2_ and CO Binding to Metalloporphyrins and Heme Proteins. Proc. Natl. Acad. Sci. USA.

[3] Collman J.P., Boulatov R., Sunderland C.J., Fu L. (2004). Functional Analogues of Cytochrome c Oxidase, Myoglobin, and Hemoglobin. Chem. Rev..

[4] D’Souza F. (2015). Expanded Porphyrins: More Confusion All the Time. Angew. Chem. Int. Ed..

[5] de la Torre G., Claessens C.G., Torres T. (2007). Phthalocyanines: Old Dyes, New Materials. Putting Color in Nanotechnology. Chem. Commun..

[6] Beletskaya I., Tyurin V.S., Tsivadze A.Y., Guilard R., Stern C. (2009). Supramolecular Chemistry of Metalloporphyrins. Chem. Rev..

[7] Mack J., Kobayashi N. (2011). Low Symmetry Phthalocyanines and Their Analogues. Chem. Rev..

[8] Glowacka-Sobotta A., Wrotynski M., Kryjewski M., Sobotta L., Mielcarek J. (2019). Porphyrinoids in Photodynamic Diagnosis and Therapy of Oral Diseases. J. Porphyr. Phthalocyanines.

[9] Sobotta L., Skupin-Mrugalska P., Piskorz J., Mielcarek J. (2019). Porphyrinoid Photosensitizers Mediated Photodynamic Inactivation against Bacteria. Eur. J. Med. Chem..

[10] Kryjewski M., Goslinski T., Mielcarek J. (2015). Functionality Stored in the Structures of Cyclodextrin–Porphyrinoid Systems. Coord. Chem. Rev..

[11] Skupin-Mrugalska P., Piskorz J., Gośliński T., Mielcarek J., Konopka K., Düzgüneş N. (2013). Current Status of Liposomal Porphyrinoid Photosensitizers. Drug Discov. Today.

[12] Claessens C.G., Hahn U., Torres T. (2008). Phthalocyanines: From Outstanding Electronic Properties to Emerging Applications. Chem. Rec..

[13] de la Torre G., Vázquez P., Agulló-López F., Torres T. (2004). Role of Structural Factors in the Nonlinear Optical Properties of Phthalocyanines and Related Compounds. Chem. Rev..

[14] Szymczak J., Rębiś T., Mielcarek J., Kryjewski M. (2022). Electrochemical, Spectrochemical and Catalytical Properties of Cobalt(II) Phthalocyanine Regioisomers Studies. Synth. Met..

[15] Koczorowski T., Ber J., Sokolnicki T., Teubert A., Szczolko W., Goslinski T. (2020). Electrochemical and Catalytic Assessment of Peripheral Bromoaryl-Substituted Manganese and Iron Porphyrazines. Dye. Pigment..

[16] Breslow R., Breslow R. (2005). Artificial Enzymes. Artificial Enzymes.

[17] Fang Z., Breslow R. (2005). A Thiolate Ligand on a Cytochrome P-450 Mimic Permits the Use of Simple Environmentally Benign Oxidants for Biomimetic Steroid Hydroxylation in Water. Bioorg. Med. Chem. Lett..

[18] Musial J., Krakowiak R., Frankowski R., Spychala M., Dlugaszewska J., Dobosz B., Bendzinska-Berus W., Krzyminiewski R., Tykarska E., Zgoła-Grześkowiak A. (2022). Simple Modification of Titanium(IV) Oxide for the Preparation of a Reusable Photocatalyst. Mater. Sci. Eng. B.

[19] Krakowiak R., Musial J., Frankowski R., Spychala M., Mielcarek J., Dobosz B., Krzyminiewski R., Sikorski M., Bendzinska-Berus W., Tykarska E. (2020). Phthalocyanine-Grafted Titania Nanoparticles for Photodegradation of Ibuprofen. Catalysts.

[20] Biesaga M. (2000). Porphyrins in Analytical Chemistry. A Review. Talanta.

[21] Pirouzmand M., Amini M.M., Safari N. (2007). Microwave-Assisted Immobilization of Metallophthalocyanines in Mesoporous Channels of MCM-41. J. Porphyr. Phthalocyanines.

[22] Beck J.S., Vartuli J.C., Roth W.J., Leonowicz M.E., Kresge C.T., Schmitt K.D., Chu C.T.W., Olson D.H., Sheppard E.W. (1992). A New Family of Mesoporous Molecular Sieves Prepared with Liquid Crystal Templates. J. Am. Chem. Soc..

[23] Bagshaw S.A., Prouzet E., Pinnavaia T.J. (1995). Templating of Mesoporous Molecular Sieves by Nonionic Polyethylene Oxide Surfactants. Science.

[24] Biswas K., Ray J.C., Choi J.-S., Ahn W.-S. (2008). Morphology Control of MSU-1 Silica Particles. J. Non-Cryst. Solids.

[25] Stevens W.J.J., Lebeau K., Mertens M., Van Tendeloo G., Cool P., Vansant E.F. (2006). Investigation of the Morphology of the Mesoporous SBA-16 and SBA-15 Materials. J. Phys. Chem. B.

[26] Zhao M., Feng M., Huo J.J. (1998). Triblock Copolymer Syntheses of Mesoporous Silica with Periodic 50 to 300 Angstrom Pores. Science.

[27] Sorokin A.B. (2013). Phthalocyanine Metal Complexes in Catalysis. Chem. Rev..

[28] Nodzewska A., Wadolowska A., Watkinson M. (2019). Recent Advances in the Catalytic Oxidation of Alkene and Alkane Substrates Using Immobilized Manganese Complexes with Nitrogen Containing Ligands. Coord. Chem. Rev..

[29] Calvete M.J.F., Silva M., Pereira M.M., Burrows H.D. (2013). Inorganic Helping Organic: Recent Advances in Catalytic Heterogeneous Oxidations by Immobilised Tetrapyrrolic Macrocycles in Micro and Mesoporous Supports. RSC Adv..

[30] Franco F., Rettenmaier C., Jeon H.S., Cuenya B.R. (2020). Transition Metal-Based Catalysts for the Electrochemical CO2 Reduction: From Atoms and Molecules to Nanostructured Materials. Chem. Soc. Rev..

[31] Zhang H., Liu G., Shi L., Ye J. (2018). Single-Atom Catalysts: Emerging Multifunctional Materials in Heterogeneous Catalysis. Adv. Energy Mater..

[32] Liang H.-W., Brüller S., Dong R., Zhang J., Feng X., Müllen K. (2015). Molecular Metal–Nx Centres in Porous Carbon for Electrocatalytic Hydrogen Evolution. Nat. Commun..

[33] Zhang H., Wei J., Dong J., Liu G., Shi L., An P., Zhao G., Kong J., Wang X., Meng X. (2016). Efficient Visible-Light-Driven Carbon Dioxide Reduction by a Single-Atom Implanted Metal-Organic Framework. Angew. Chem. Int. Ed..

[34] Huang C.-X., Lv S.-Y., Li C., Peng B., Li G., Yang L.-M. (2022). Single-Atom Catalysts Based on Two-Dimensional Metalloporphyrin Monolayers for Ammonia Synthesis under Ambient Conditions. Nano Res..

[35] Xiao M., Zhu J., Li G., Li N., Li S., Cano Z.P., Ma L., Cui P., Xu P., Jiang G. (2019). A Single-Atom Iridium Heterogeneous Catalyst in Oxygen Reduction Reaction. Angew. Chem. Int. Ed..

[36] Liu C.-J., Li S.-G., Pang W.-Q., Che C.-M. (1997). Ruthenium Porphyrin Encapsulated in Modified Mesoporous Molecular Sieve MCM-41 for Alkene Oxidation. Chem. Commun..

[37] Ernst S., Selle M. (1999). Immobilization and Catalytic Properties of Perfluorinated Ruthenium Phthalocyanine Complexes in MCM-41-Type Molecular Sieves. Microporous Mesoporous Mater..

[38] Bedioui F. (1995). Zeolite-Encapsulated and Clay-Intercalated Metal Porphyrin, Phthalocyanine and Schiff-Base Complexes as Models for Biomimetic Oxidation Catalysts: An Overview. Coord. Chem. Rev..

[39] Sabour B., Mehrani K., Mehrani A., Peyrovi M.H. (2013). Immobilization of Metalloporphyrin on Functionalized MCM-48 Nanoporous Silica as a Catalyst in Oxidation of Cyclohexene: The Effects of Nanoporous Structure of MCM-48 on Its Catalytic Efficiency. J. Inorg. Organomet. Polym. Mater..

[40] Zanjanchi M.A., Ebrahimian A., Arvand M. (2010). Sulphonated Cobalt Phthalocyanine–MCM-41: An Active Photocatalyst for Degradation of 2,4-Dichlorophenol. J. Hazard. Mater..

[41] Zhang Y.-P., Xue K.-C., Zhang W.-P., Song C., Zhang R.-L., Zhao J.-S. (2013). Synthesis and Catalytic Performance of MCM-41 Modified with Tetracarboxylphthalocyanine. Chem. Pap..

[42] Sorokin A.B., Tuel A. (2000). Metallophthalocyanine Functionalized Silicas: Catalysts for the Selective Oxidation of Aromatic Compounds. Catal. Today.

[43] Tanamura Y., Uchida T., Teramae N., Kikuchi M., Kusaba K., Onodera Y. (2001). Ship-in-a-Bottle Synthesis of Copper Phthalocyanine Molecules within Mesoporous Channels of MCM-41 by a Chemical Vapor Deposition Method. Nano Lett..

[44] Najafian A., Rabbani M., Rahimi R., Deilamkamar M., Maleki A. (2015). Synthesis and Characterization of Copper Porphyrin into SBA-16 through “Ship in a Bottle” Method: A Catalyst for Photo Oxidation Reaction under Visible Light. Solid State Sci..

[45] Li J., Guo L., Shi J. (2010). Stepwise in Situ Synthesis and Characterization of Metallophthalocyanines@mesoporous Matrix SBA-15 Composites. Phys. Chem. Chem. Phys..

[46] Radha Rani V., Radha Kishan M., Kulkarni S.J., Raghavan K.V. (2005). Immobilization of Metalloporphyrin Complexes in Molecular Sieves and Their Catalytic Activity. Catal. Commun..

[47] Nakagaki S., KB Ferreira G., Marcalb A.L., Ciuffi K.J. (2014). Metalloporphyrins Immobilized on Silica and Modified Silica as Catalysts in Heterogeneous Processes. Curr. Org. Synth..

[48] Battioni P., Lallier J.-P., Barloy L., Mansuy D. (1989). Mono-Oxygenase-like Oxidation of Hydrocarbons Using Supported Manganese?Porpnyrin Catalysts: Beneficial Effects of a Silica Support for Alkane Hydroxylation. J. Chem. Soc. Chem. Commun..

[49] Bertrand E.M., Keddis R., Groves J.T., Vetriani C., Austin R.N. (2013). Identity and Mechanisms of Alkane-Oxidizing Metalloenzymes from Deep-Sea Hydrothermal Vents. Front. Microbiol..

[50] Wang V.C.-C., Maji S., Chen P.P.-Y., Lee H.K., Yu S.S.-F., Chan S.I. (2017). Alkane Oxidation: Methane Monooxygenases, Related Enzymes, and Their Biomimetics. Chem. Rev..

[51] Połtowicz J., Pamin K., Matachowski L., Serwicka E.M., Mokaya R., Xia Y., Olejniczak Z. (2006). Oxidation of Cyclooctane over Mn(TMPyP) Porphyrin-Exchanged Al,Si-Mesoporous Molecular Sieves of MCM-41 and SBA-15 Type. Catal. Today.

[52] Pinto V.H.A., Rebouças J.S., Ucoski G.M., de Faria E.H., Ferreira B.F., Silva San Gil R.A., Nakagaki S. (2016). Mn Porphyrins Immobilized on Non-Modified and Chloropropyl-Functionalized Mesoporous Silica SBA-15 as Catalysts for Cyclohexane Oxidation. Appl. Catal. Gen..

[53] Thielemann J.P., Girgsdies F., Schlögl R., Hess C. (2011). Pore Structure and Surface Area of Silica SBA-15: Influence of Washing and Scale-Up. Beilstein J. Nanotechnol..

[54] Ucoski G.M., Pinto V.H.A., DeFreitas-Silva G., Rebouças J.S., Marcos da Silva R., Mazzaro I., Nunes F.S., Nakagaki S. (2018). Manganese Porphyrins Immobilized on Magnetic SBA-15 Mesoporous Silica as Selective and Efficient Catalysts for Cyclic and Linear Alkane Oxidation. Microporous Mesoporous Mater..

[55] Armengol E., Corma A., Fornés V., García H., Primo J. (1999). Cu^2+^-Phthalocyanine and Co^2+^-Perfluorophthalocyanine Incorporated inside Y Faujasite and Mesoporous MCM-41 as Heterogeneous Catalysts for the Oxidation of Cyclohexane. Appl. Catal. Gen..

[56] Karandikar P., Chandwadkar A., Agashe M., Ramgir N., Sivasanker S. (2006). Liquid Phase Oxidation of Alkanes Using Cu/Co-Perchlorophthalocyanine Immobilized MCM-41 under Mild Reaction Conditions. Appl. Catal. Gen..

[57] Sato K., Aoki M., Noyori R. (1998). A “Green” Route to Adipic Acid: Direct Oxidation of Cyclohexenes with 30 Percent Hydrogen Peroxide. Science.

[58] Li Z., Xia C.-G., Zhang X.-M. (2002). Preparation and Catalysis of DMY and MCM-41 Encapsulated Cationic Mn (III)–Porphyrin Complex. J. Mol. Catal. Chem..

[59] Costa A.A., Ghesti G.F., de Macedo J.L., Braga V.S., Santos M.M., Dias J.A., Dias S.C.L. (2008). Immobilization of Fe, Mn and Co Tetraphenylporphyrin Complexes in MCM-41 and Their Catalytic Activity in Cyclohexene Oxidation Reaction by Hydrogen Peroxide. J. Mol. Catal. Chem..

[60] Zimowska M., Michalik-Zym A., Połtowicz J., Bazarnik M., Bahranowski K., Serwicka E.M. (2007). Catalytic Oxidation of Cyclohexene over Metalloporphyrin Supported on Mesoporous Molecular Sieves of FSM-16 Type—Steric Effects Induced by Nanospace Constraints. Catal. Today.

[61] Kalilur Rahiman A., Rajesh K., Shanmuga Bharathi K., Sreedaran S., Narayanan V. (2006). Manganese(III) Porphyrin-Encapsulated Ti,Si-Mesoporous Molecular Sieves as Heterogeneous Catalysts for the Epoxidation of Alkenes. Appl. Catal. Gen..

[62] Kalilur Rahiman A., Shanmuga Bharathi K., Sreedaran S., Rajesh K., Narayanan V. (2009). Cationic Vanadyl Porphyrin-Encapsulated Mesoporous Al/V-MCM-41 as Heterogeneous Catalysts for the Oxidation of Alkenes. Inorg. Chim. Acta.

[63] Zanardi F.B., Barbosa I.A., de Sousa Filho P.C., Zanatta L.D., da Silva D.L., Serra O.A., Iamamoto Y. (2016). Manganese Porphyrin Functionalized on Fe3O4@nSiO2@MCM-41 Magnetic Composite: Structural Characterization and Catalytic Activity as Cytochrome P450 Model. Microporous Mesoporous Mater..

[64] Hajian R., Ehsanikhah A. (2018). Manganese Porphyrin Immobilized on Magnetic MCM-41 Nanoparticles as an Efficient and Reusable Catalyst for Alkene Oxidations with Sodium Periodate. Chem. Phys. Lett..

[65] Khalili N.R., Rahimi R., Rabbani M. (2013). Immobilized Metalloporphyrins in Mesoporous MCM-48 as Efficient and Selective Heterogeneous Catalysts for Oxidation of Cyclohexene. Mon. Für. Chem. Chem. Mon..

[66] Farokhi A., Hosseini Monfared H. (2017). Highly Efficient Asymmetric Epoxidation of Olefins with a Chiral Manganese-Porphyrin Covalently Bound to Mesoporous SBA-15: Support Effect. J. Catal..

[67] Berijani K., Hosseini-Monfared H. (2018). Collaborative Effect of Mn-Porphyrin and Mesoporous SBA-15 in the Enantioselective Epoxidation of Olefins with Oxygen. Inorg. Chim. Acta.

[68] Farahmand S., Ghiaci M. (2019). Highly Selective Allylic Oxidation of Cyclohexene to 2-Cyclohexen-1-One under Mild Conditions over Vanadyl-Porphyrin Implanted onto the Amino-Functionalized SBA-15. Microporous Mesoporous Mater..

[69] Masteri-Farahani M., Rahimi M., Hosseini M.-S. (2020). Heterogenization of Porphyrin Complexes within the Nanocages of SBA-16: New Efficient and Stable Catalysts for the Epoxidation of Olefins. Colloids Surf. Physicochem. Eng. Asp..

[70] Saikia L., Srinivas D. (2009). Redox and Selective Oxidation Properties of Mn Complexes Grafted on SBA-15. Catal. Today.

[71] Tüysüz H., Galilea J.L., Schüth F. (2009). Highly Diluted Copper in a Silica Matrix as Active Catalyst for Propylene Oxidation to Acrolein. Catal. Lett..

[72] Pirouzmand M., Amini M.M., Safari N. (2008). Immobilization of Iron Tetrasulfophthalocyanine on Functionalized MCM-48 and MCM-41 Mesoporous Silicas: Catalysts for Oxidation of Styrene. J. Colloid Interface Sci..

[73] Nur H., Hamid H., Endud S., Hamdan H., Ramli Z. (2006). Iron-Porphyrin Encapsulated in Poly(Methacrylic Acid) and Mesoporous Al-MCM-41 as Catalysts in the Oxidation of Benzene to Phenol. Mater. Chem. Phys..

[74] Yang F., Gao S., Xiong C., Wang H., Chen J., Kong Y. (2015). Coordination of Manganese Porphyrins on Amino-Functionalized MCM-41 for Heterogeneous Catalysis of Naphthalene Hydroxylation. Chin. J. Catal..

[75] Farahmand S., Ghiaci M., Asghari S. (2021). Oxo-Vanadium (IV) Phthalocyanine Implanted onto the Modified SBA-15 as a Catalyst for Direct Hydroxylation of Benzene to Phenol in Acetonitrile-Water Medium: A Kinetic Study. Chem. Eng. Sci..

[76] Huang C., Liu R., Yang W., Zhang C., Zhu H. (2017). Iron(II) Phthalocyanine Immobilized SBA-15 Catalysts: Preparation, Characterization and Application for Toluene Selective Aerobic Oxidation. Inorg. Chim. Acta.

[77] Zhu Q., Maeno S., Nishimoto R., Miyamoto T., Fukushima M. (2014). Oxidative Degradation of Pentabromophenol in the Presence of Humic Substances Catalyzed by a SBA-15 Supported Iron-Porphyrin Catalyst. J. Mol. Catal. Chem..

[78] Sirotin S.V., Tolbin A.Y., Moskovskaya I.F., Abramchuk S.S., Tomilova L.G., Romanovsky B.V. (2010). Heterogenized Fe(III) Phthalocyanine: Synthesis, Characterization and Application in Liquid-Phase Oxidation of Phenol. J. Mol. Catal. Chem..

[79] Shen C., Wen Y., Shen Z., Wu J., Liu W. (2011). Facile, Green Encapsulation of Cobalt Tetrasulfophthalocyanine Monomers in Mesoporous Silicas for the Degradative Hydrogen Peroxide Oxidation of Azo Dyes. J. Hazard. Mater..

[80] Molinari A., Maldotti A., Bratovcic A., Magnacca G. (2011). Fe(III)-Porphyrin Heterogenized on MCM-41: Matrix Effects on the Oxidation of 1,4-Pentanediol. Catal. Today.

[81] Najafian A., Rahimi R., Zargari S., Mahjoub-Moghaddas M., Nazemi A. (2015). Synthesis and Photocatalytic Activity of V-Doped Mesoporous TiO2 Photosensitized with Porphyrin Supported by SBA-15. Res. Chem. Intermed..

[82] Silva M., Calvete M.J.F., Gonçalves N.P.F., Burrows H.D., Sarakha M., Fernandes A., Ribeiro M.F., Azenha M.E., Pereira M.M. (2012). Zinc(II) Phthalocyanines Immobilized in Mesoporous Silica Al-MCM-41 and Their Applications in Photocatalytic Degradation of Pesticides. J. Hazard. Mater..

[83] Gutmann B., Cantillo D., Kappe C.O. (2015). Continuous-Flow Technology—A Tool for the Safe Manufacturing of Active Pharmaceutical Ingredients. Angew. Chem. Int. Ed..

[84] Anastas P., Eghbali N. (2010). Green Chemistry: Principles and Practice. Chem. Soc. Rev..

